# Biodegradation of bio-sourced and synthetic organic electronic materials towards green organic electronics

**DOI:** 10.1038/s41467-021-23227-4

**Published:** 2021-05-26

**Authors:** Eduardo Di Mauro, Denis Rho, Clara Santato

**Affiliations:** 1 Département de Génie Physique, Polytechnique Montréal, Montréal, QC Canada; 2 Aquatic and Crop Resource Development, National Research Council Canada, Montréal, QC Canada

**Keywords:** Environmental impact, Electronic devices

## Abstract

Ubiquitous use of electronic devices has led to an unprecedented increase in related waste as well as the worldwide depletion of reserves of key chemical elements required in their manufacturing. The use of biodegradable and abundant organic (carbon-based) electronic materials can contribute to alleviate the environmental impact of the electronic industry. The pigment eumelanin is a bio-sourced candidate for environmentally benign (green) organic electronics. The biodegradation of eumelanin extracted from cuttlefish ink is studied both at 25 °C (mesophilic conditions) and 58 °C (thermophilic conditions) following ASTM D5338 and comparatively evaluated with the biodegradation of two synthetic organic electronic materials, namely copper (II) phthalocyanine (Cu–Pc) and polyphenylene sulfide (PPS). Eumelanin biodegradation reaches 4.1% (25 °C) in 97 days and 37% (58 °C) in 98 days, and residual material is found to be without phytotoxic effects. The two synthetic materials, Cu–Pc and PPS, do not biodegrade; Cu–Pc brings about the inhibition of microbial respiration in the compost. PPS appears to be potentially phytotoxic. Finally, some considerations regarding the biodegradation test as well as the disambiguation of “biodegradability” and “bioresorbability” are highlighted.

## Abstract (web summary)

Waste build-up from organic electronic components is a major environmental issue; biodegradable electronic materials could be a solution to this. Here, the authors report on the biodegradation of bio-sourced and synthetic electronic materials in industrial compost at different temperatures.

## Introduction

Electronic equipment has become ubiquitous in our everyday life. The increase of waste electrical and electronic equipment (WEEE), 53.6 Mt worldwide in 2019^1^, and the depletion of chemical elements of prime importance in the electronic industry, such as indium and gallium, have put mounting pressure on the environment^2^. Limited attention has been dedicated to possible EEE end-of-life scenarios. The focus has been on device performance, as the technological advances of the last decades confirm. Refurbishment and recycling of electronic devices have been identified, among others, as economically and environmentally viable solutions to deal with WEEE^1^. Besides that, a promising route towards achieving sustainable (green) electronics is based on the use of abundant materials (including biomolecules extracted from biomass feedstock), novel production schemes involving non-toxic solvents, and eco-design of devices that includes biodegradation at end of life^2^. Organic (carbon-based) electronic materials are ideal candidates to explore such a route^2–5^.

Organic electronics is based on molecules and polymers that feature electronic conjugation by means of alternating single and double carbon–carbon bonds^6^. Organic electronic devices such as organic light-emitting diodes, photovoltaic cells, field-effect transistors, and sensors have been demonstrated^7,8^. They stand out for their mechanical properties, such as flexibility, rollability, and stretchability, which are of great interest for applications such as wearable electronics, imperceptible electronics, and smart packaging^9^. Being solution-processable (printable), organic electronic devices feature lower embodied energy, i.e., energy spent in the production phase and stored in inner constituents, with respect to their inorganic counterparts, processed by high-vacuum and high-temperature techniques^2^. Our vision is to develop eco-friendly organic electronic materials and devices with reduced environmental impact, designed and certified to be compostable at their end of life.

All over the world, municipalities have adopted composting technology as an environmentally friendly approach to managing solid organic wastes. Composting offers many advantages. It successfully diverts organic wastes from disposal in landfills^10^ and converts them into simple components such as CO_2_, H_2_O, minerals, and humic-like compost. This bioprocess is driven by a community of living organisms, i.e., compost microbiota, whose rate of activity is influenced by environmental parameters, such as oxygen level, pH, temperature, water activity (*a*_*w*_), *C*/*N* ratio, compost granulometry, and water content^11,12^. Composting reaches its optimal degradation efficiency when the conditions are met for a thermophilic phase (50–70 °C) to take place. The particle size of the material to be composted is another important parameter.

To assess biodegradability in composting conditions, several standard tests are available for the industrial sector of disposable plastics for food packaging and serving. Examples of such standards are CAN/BNQ 0017-088/2010^13^ in Canada, ASTM D6400^14^ in the USA, EN 13432^15^, and EN 14995^16^ in the European Union. Poly-ε-caprolactone (PCL) and polylactic acid (PLA) are among the most important plastics produced with a high degree of degradability under composting conditions^17^. Conversely, as green organic electronics is an emerging field, limited attention has been given to the biodegradability of materials and devices for organic electronics: in fact, no national or international standard exists to evaluate their biodegradability in municipal waste treatment facilities.

**Fig. 1:**
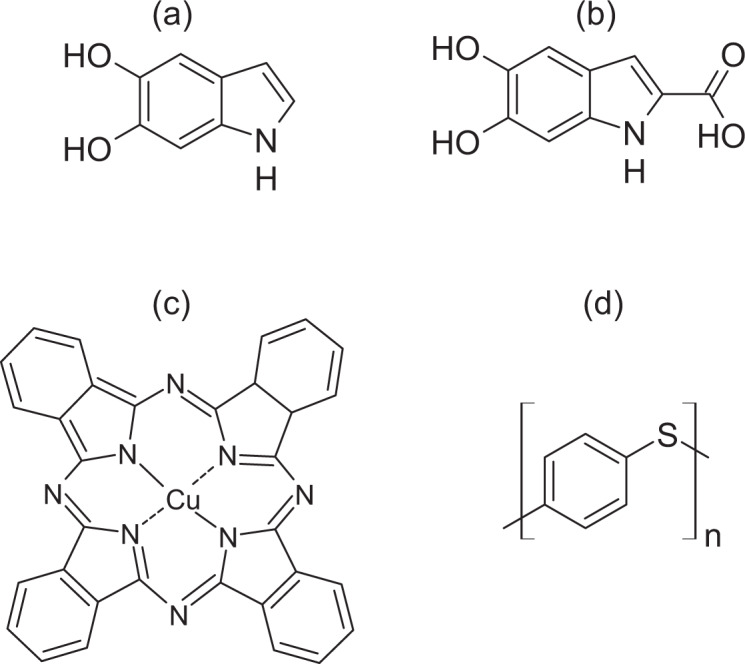
Molecular structures of the organic electronic materials investigated. **a** 5,6-dihydroxyindole (DHI) and (**b**) 5,6-dihydroxyindole-2-carboxylic acid (DHICA), the building blocks of eumelanin. **c** Cu (II) phthalocyanine (Cu–Pc), and (**d**) monomer of polyphenylene sulfide (PPS).

Melanins represent a vast category of pigments ubiquitous in nature and noticeable in our daily life: from human skin color to black spots on moldy bread, from black incrustations on shower curtains to dark parts of banana peels^18,19^. Eumelanin is the subcategory of black-brown melanin pigments that originates from the oxidative polymerization of L-dopa^20^, mostly present in animals, whose building blocks are 5,6-dihydroxyindole (DHI) and 5,6-dihydroxyindole-2-carboxylic acid (DHICA) (Fig. 1). Relevant properties of eumelanin are its broad UV–visible absorption, free-radical scavenging, and hygroscopicity^21^. Importantly, melanin is a chemically and thermally stable material^22,23^. Eumelanin is an interesting candidate for green organic electronics because its molecular structure features conjugation and redox-active quinone groups^24^. Hydration-dependent electrical conduction has been observed in eumelanin pellets and films, and electronic transport has been recently reported by our group in dry natural eumelanin extracted from the ink sac of cuttlefish (Sepia Melanin) pellets^25–27^. Eumelanin-based supercapacitors^28^ and batteries^29^ as well as organic electrochemical transistors based on eumelanin as the gating medium^30^ have been successfully developed. Considering its abundance, non-toxicity, biocompatibility^31^, biodegradability potential (the subject of this work), and the option of producing it in large amounts^32^, eumelanin is an environmentally friendly, prototypically benign material suitable to explore the potential of sustainable organic electronics and its powering elements.

Biodegradability assessments on synthetic eumelanin (dopa-melanin in a mixed bacterial culture^33^ and tyrosine-melanin mixed in soil^34^) and natural eumelanin (human hair^35^ in loamy soil) showed negligible biodegradation. Eumelanin was reported to be present in the orbit of a 54 My fish fossil^36^. Conversely, the fungus *Aspergillus fumigatus* from a soil sample degraded different types of natural eumelanins (human hair, human skin, banana peel, insects, and octopus ink)^37–39^ and synthetic tyrosine- and dopa-melanin^38^.

In this work, we report on the biodegradability of Sepia Melanin blended with industrial compost obtained from a municipal solid waste treatment facility, under mesophilic (25 °C) and thermophilic (58 °C) conditions. These conditions are quite different from biochemical turnover in vivo and biodegradation ex vivo. Our work does not deal with biodegradability as generic unspecified disappearance in natural ecosystems such as soils, lakes, marine environments. It specifically deals with biodegradability in industrial composting conditions, i.e., man-made ecosystems in which municipal wastes can be disposed of. Prior to the biodegradability studies, the extracted biopigment was characterized by infrared spectroscopy (IR), thermogravimetric analysis (TGA), nuclear activation analysis (NAA), as well as elemental analyses of total carbon and total inorganic carbon (TIC).

The biodegradability test in composting conditions included two synthetic (non-bio-sourced) materials representative of the two classes of organic electronic materials: those that can be processed by thermal evaporation and those that are solution-processable^40^. These materials were copper (II) phthalocyanine (Cu–Pc) and polyphenylene sulfide (PPS). Cu–Pc is a molecular dye, with semiconducting properties employed in organic field-effect transistors and photovoltaic cells^41–43^. PPS is a solution-processable polymer, whose electrical conductivity can be increased by 16 orders of magnitude by chemical doping, applied in photovoltaic cells^44^.

The scientific problem we address is the compostability of one bio-sourced and two conventional synthetic electronic ingredients, using the ASTM D5338^45^ protocol under operating conditions typical of an industrial composting facility, i.e., thermophilic (58 °C) and aerobic conditions.

Last but not least, eumelanin is also bioresorbable, as demonstrated by studies using the biopigment in body implants^31,46^. Unlike biodegradability in compost, bioresorption is a process by which materials in contact with the physiological environment (aqueous, body fluids) are degraded and their by-products are eliminated or completely bioresorbed by the recipient host^47,48^. In most of the work claiming biodegradability of organic electronic devices, such devices are put in contact with aqueous electrolytic solutions similar to human body fluids (bioresorbability conditions)^49,50^. Our work clarifies the difference between biodegradability in composting conditions (man-made ecosystem), a conceivable end-of-life scenario for (some) organic electronic waste^10^, and bioresorbability^47^, relevant for transient electronics and/or organic electronic materials to be employed in body implants^51^.

## Results

**Fig. 2:**
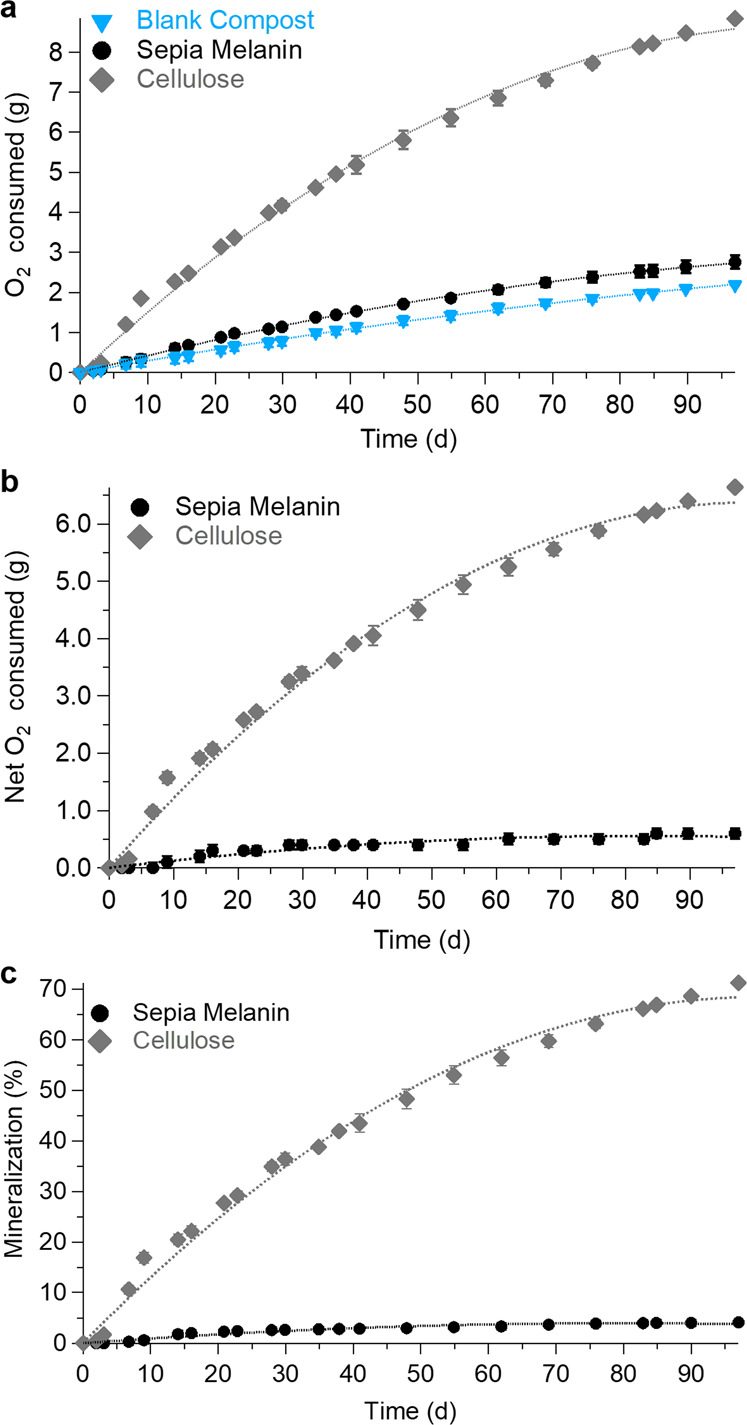
Biodegradation under mesophilic conditions (25 °C). **a** Cumulative O_2_ consumed: blank compost, Sepia Melanin, and cellulose. **b** Net O_2_ consumed by Sepia Melanin and cellulose, with respect to the O_2_ consumption of the blank compost. **c** Mineralization of Sepia Melanin and cellulose. Error bars = standard deviation (*n* = 3 for Sepia Melanin, *n* = 2 for blank compost and cellulose).

Throughout the incubation period, the respiration activity of the compost microbiota was monitored by electrolytic respirometers, measuring the amount of O_2_ consumed. The cumulative O_2_ consumed was comparatively measured for Sepia Melanin and cellulose blended with the compost, i.e., the test material and the positive control, respectively (Fig. 2a). A blank compost was used to obtain the background respiration activity (Fig. 2a). The O_2_ consumed by the compost microbiota increased steadily over the entire incubation period (97 days). Approximately 70% of the final value was consumed during the first 50 days (Δ*t*_0-50_), while at the end of the biodegradability test, the O_2_ respiratory levels for Sepia Melanin and cellulose were 2731 and 8835 mg, respectively. These levels are 1.3× and 4.0× higher than the baseline (2194 mg). After subtracting the cumulative O_2_ respired by the blank compost from the cumulative O_2_ respired by Sepia Melanin and cellulose (Fig. 2b), using Eqs. (2) and (3), the mineralization levels were calculated (Fig. 2c). These levels were 4.1 ± 0.7% (standard deviation) for Sepia Melanin and 71.2 ± 0.2% for cellulose, at the end of the incubation period.

**Fig. 3:**
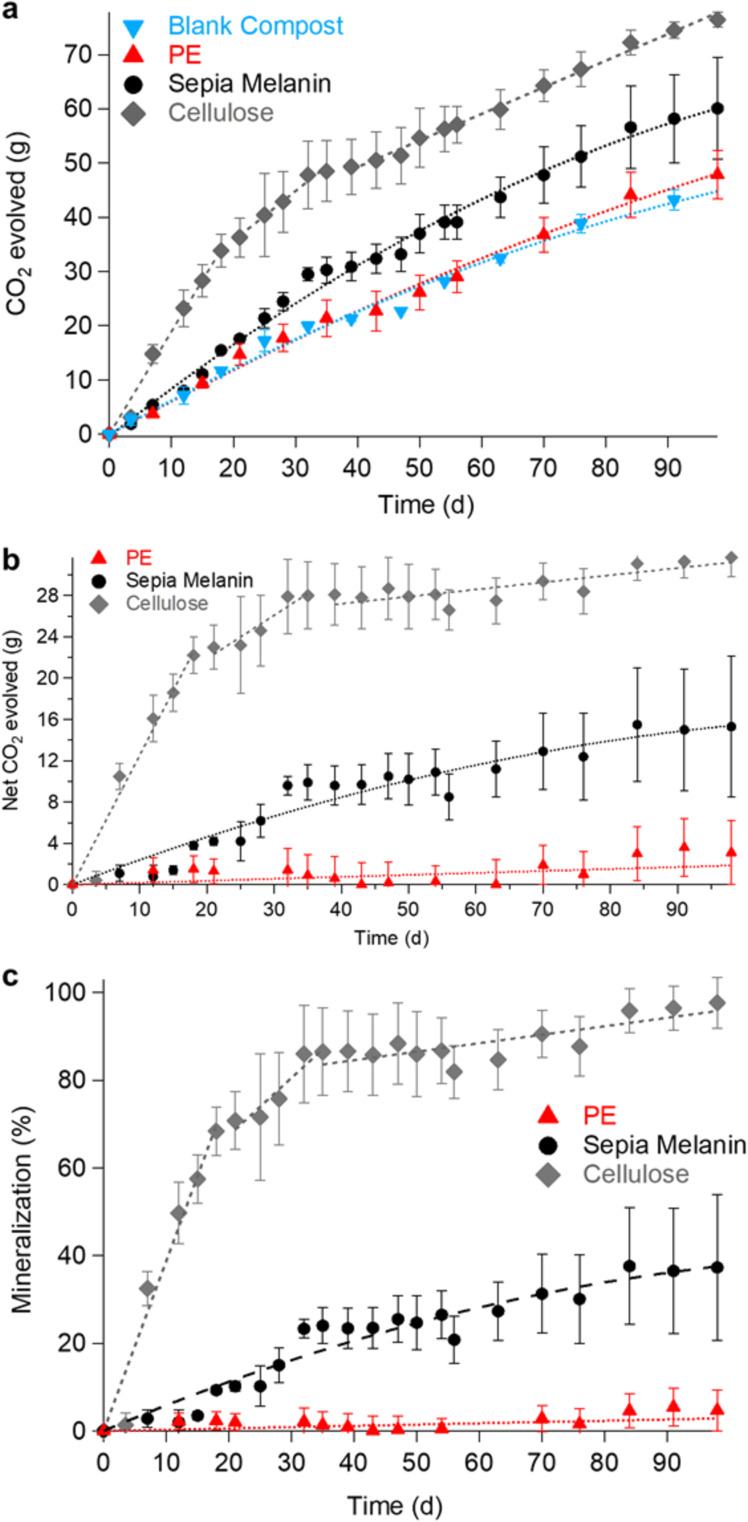
Biodegradation of Sepia Melanin in composting conditions (58 °C). **a** Cumulative CO_2_ evolved from blank compost, polyethylene (PE), Sepia Melanin, and cellulose. For the blank compost and PE, only half of the points are shown for the sake of clarity, as they overlapped (Supplementary Fig. 3). **b** Net CO_2_ evolved from PE, Sepia Melanin, and cellulose. **c** Mineralization of PE, Sepia Melanin, and cellulose. Error bars = standard deviation (*n* = 3 for PE and Cellulose, *n* = 2 for blank compost and Sepia Melanin).

The biodegradability test under thermophilic conditions (58 °C, composting conditions with a constant supply of O_2_ via a flow of humid air) entailed the monitoring of the respiration activity of the compost microbiota by the measurement of the CO_2_ evolved. The cumulative CO_2_ evolved was measured for the blank compost (background), polyethylene (PE) pellets buried in compost (negative control), cellulose blended with compost (positive control) and Sepia Melanin blended with compost (Fig. 3a). The net cumulative CO_2_ produced was obtained by the subtraction of the CO_2_ evolved from the blank compost (Fig. 3b). From the net cumulative CO_2_ evolved, by means of Eqs. (2) and (3), the mineralization levels of cellulose, PE, and Sepia Melanin were calculated (Fig. 3c). After 98 days, the mineralization level of PE (negative control) was zero compared to 97 ± 6% for cellulose (positive control) while Sepia Melanin reached a mineralization level of 37 ± 17% (Fig. 3c). Temperature is a major factor that influences the rate of biodegradation of melanin; it is 9× higher at 58 °C than at 25 °C (Table 1). Despite the rate of biodegradation being higher, it is 12× lower than the rate of biodegradation of cellulose, in the first 21 days (Δ*t*_0-21_). During the next 77 days, after plummeting by a factor of 20, the rate of cellulose biodegradation decreases gradually and steadily to a low level, most probably due to complete biodegradation. In that same period, the biodegradation of eumelanin continued, but at an even lower rate than during the first period (Fig. 3c).

**Table 1:** Biodegradation summary.

Compost + test material	Biodegradability test (25 °C) after 97 days			Biodegradability test (58 °C) after 98 days		
	Cumulative O_2_ consumed (mg)	O_2_ consumption rate (mg/d)	Mineralization (%)	Cumulative CO_2_ evolved (mg)	CO_2_ respired rate (mg/d)	Mineralization (%)
Blank compost (Background)	2194 ± 3	22.62 ± 0.03	–	44,771 ± 2387	457 ± 24	–
Polyethylene (Negative control)	nd	nd	nd	47,864 ± 4455	488 ± 45	0
Cellulose (Positive control)	8835 ± 23	91.08 ± 0.23	71.2 ± 0.2	76,454 ± 1400	780 ± 14	98 ± 6
Sepia Melanin	2759 ± 156	28.44 ± 1.61	4.1 ± 0.7	60,099 ± 9385	613 ± 96	37 ± 17
Copper (II) phthalocyanine	nd	nd	nd	39,190 ± 885	400 ± 9	0
Polyphenylene sulfide	nd	nd	nd	46,833 ± 4468	478 ± 46	0

Respiration rates under mesophilic (25 °C) and thermophilic (58 °C) conditions for various combinations of compost and test material (bio-sourced and synthetic) buried in 100 g and 250 g of compost, respectively. (–) Not applicable; (nd) not determined.

According to ASTM D6400, to be defined as aerobically biodegradable under composting conditions, test materials need to meet or exceed the 90% threshold value set by the value of the positive control (cellulose), within 45 days or at most in 180 days. Therefore, using the data obtained with a 98-day biodegradation test at 58 °C (cellulose: 97 ± 6% at 0.19%/day for Δ*t*_35–98_ and melanin: 37 ± 17% at 0.26%/d for Δ*t*_35–98_) (Supplementary Fig. 4), we project (extrapolate) that over a period of 180 days the cellulose would be completely mineralized while the level of mineralization of Sepia Melanin would reach 58%.

**Fig. 4:**
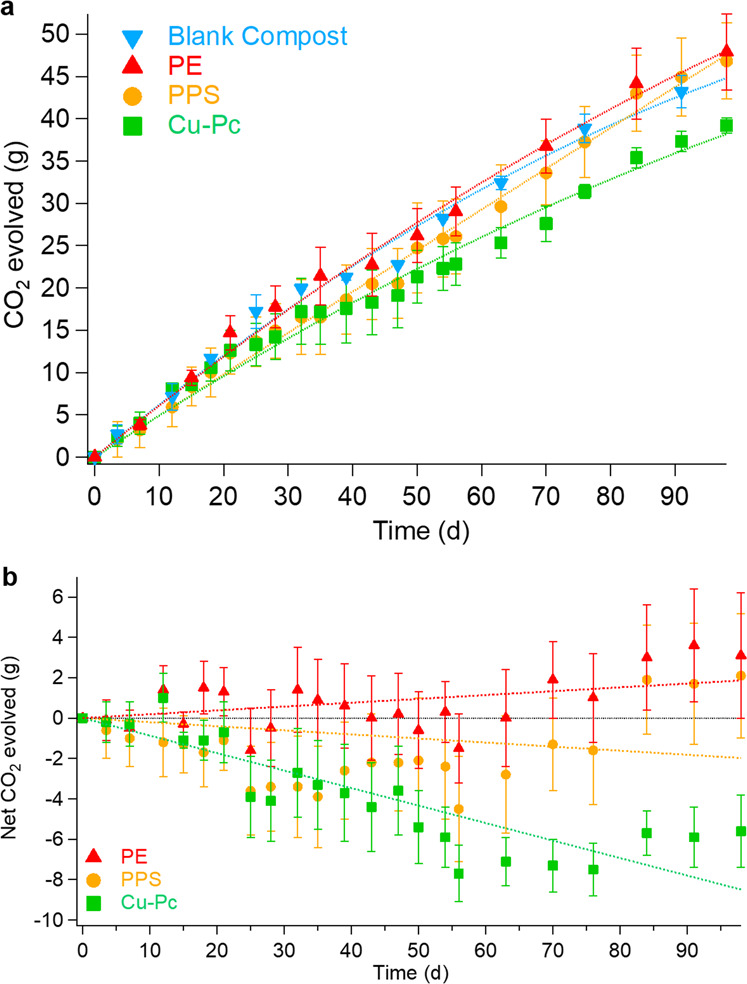
Biodegradation in composting conditions of synthetic materials. **a** Cumulative CO_2_ evolved from blank compost, PE, PPS, and Cu–Pc. **b** Net cumulative CO_2_ evolved from PE, PPS, and Cu–Pc, with respect to blank compost. Error bars = standard deviation (*n* = 3 for PE, Cu–Pc, and PPS, *n* = 2 for blank compost).

Along with the above-mentioned test materials, the biodegradability test included two synthetic organic electronic materials, namely copper(II) phthalocyanine (Cu–Pc) and PPS. The cumulative CO_2_ evolved was measured and reported along with the blank compost (background) and PE (negative control) (Fig. 4a). At day 98, the apparent respiration rate of PPS, as determined by the ratio of the total CO_2_ respired divided by the total incubation time, was 478 ± 46 mg/d. This is statistically similar to the apparent respiration rate of the blank compost (457 ± 24 mg/d) as well as of the negative control (PE) (488 ± 45 mg/d) (Fig. 4a). Regarding Cu–Pc, in the first 50 days, the respiration was similar to that of blank compost. However, from day 50, it can be seen that the CO_2_ respired does not increase as rapidly as the control. Thus, at the end of the test, there is a difference of ~−12% with the latter (Fig. 4a). The net mineralization data shows that there is no mineralization of PE (negative control) as well as 0% mineralization in the case of PPS. On the other hand, −6% is observed in the case of Cu–Pc, which suggests a possible inhibitory effect on the respiration of the microbiota of the compost.

Table 1 is a summary of the cumulative O_2_ consumed at 25 °C, the cumulative CO_2_ evolved at 58 °C, the apparent respiration rates, and the mineralization levels observed at the end of the two biodegradation tests for the bio-sourced melanin and the two synthetic materials tested.

At the end of the biodegradation test under thermophilic conditions (98 days), samples of the compost were used to conduct a terrestrial phytotoxicity test based on the determination of plant germination and biomass production. The number of plants that germinated from 20 seeds of ryegrass and the wet mass of these plants was measured after 19 days of incubation at 25 °C (Table 2). In accordance with ASTM D6400, using data obtained with the blank compost sample, a threshold value of 90% was calculated and used to statistically validate terrestrial phytotoxicity of residual materials, after 98 days of composting (Table 2). Cellulose, Sepia Melanin, and Cu–Pc showed no phytotoxicity while PE (plant biomass data only) demonstrated a potentially phytotoxic effect. In the case of PPS, both parameters are clearly below the threshold value of 90%, which suggests a possible phytotoxic effect. Furthermore, at that time, we observed none of the attributes of phytotoxicity, such as deformation, discoloration of stems and leaves, and signs of necrosis. Finally, by using the control compost as a source of active prokaryotes (fungi, actinobacteria, and bacteria), we had access to a second bioindicator of the terrestrial trophic level. Comparison of the net CO_2_ production rate of the different materials to that of the control compost (0.46 g/d) (Table 2) revealed that there was no toxic effect on the microbiota of the compost (Table 2).

**Table 2:** Phytotoxicity tests.

	Trophic level: terrestrial		
Bioindicators	Plant Seeds (ryegrass)		Microorganisms (fungi and bacteria)
Samples (at 98 days)	Seedling emergence (%)	Plant biomass (mg)	Net apparent CO_2_ production rate (g/d)
Blank compost [threshold 90%]	78 ± 15 [70%]	84 ± 28 [76 mg]	0.46 ± 0.02 [0.41 g/d]
Cellulose	89 ± 8	115 ± 30	0.78 ± 0.01
	No phytotoxicity	No phytotoxicity	No ecotoxicity
Sepia Melanin	76 ± 12	77 ± 24	0.61 ± 0.01
	No phytotoxicity	No phytotoxicity	No ecotoxicity
Polyethylene	66 ± 19	48 ± 26	0.49 ± 0.05
	No phytotoxicity	Potentially phytotoxic	No ecotoxicity
Copper (II) phthalocyanine	57 ± 13	50 ± 33	0.40 ± 0.01
	No phytotoxicity	No phytotoxicity	Potential ecotoxicity
Polyphenylene sulfide	42 ± 15	14 ± 6	0.48 ± 0.05
	Potentially phytotoxic	Potentially phytotoxic	No ecotoxicity

Seedling emergence and plant biomass in wet sandy soil (used as a “pristine” or “clean” substratum). After 19 days, the number of plants in this wet sandy soil is 20 ± 0. In addition, a second bioindicator, the compost microbiota (fungi and bacteria), representing a lower trophic level than the plants, was used. Thus, the microbial respiration rate (apparent CO_2_ production) is also reported in this table, in g/d. As recommended by ASTM, a 90% threshold value is used to validate potential phytotoxicity. Statistical analysis is detailed in SI.

## Discussion

Composting is a dynamic and robust process whose efficiency is well established for the treatment of a wide range of organic waste^52^. The biodegradation process that takes place in compost is primarily carried out by its microbiome, i.e., a community of microorganisms that includes mesophilic and thermophilic fungi, bacteria, and actinobacteria^12^. These microorganisms have evolved different enzymatic and non-enzymatic strategies^52^. The rate of biodegradation is related to abiotic factors, which include properties intrinsic to the chemical composition of the test material and its molecular and supramolecular structure, which will be discussed later. It also correlates to the environmental conditions specific to the composting process (e.g., aeration, temperature, pH, *a*_*w*_, particle surface area, and C/N ratio)^12^. Bio-sourced materials made of complex biopolymers such as lignocellulose or modified starch are biodegraded (composted) in a multi-step process. Initially, depolymerization takes place, i.e., enzymatic hydrolysis of cellulose or starch chains via, e.g., endo- and exo-glucanases^53^ and/or enzymatic oxidation of the lignin backbone via, e.g., manganese or lignin peroxidases^54,55^. Once the resulting oligomers, dimers, or monomers are released, they can be transported into the microorganisms^56^. Finally, mineralization follows, ending with the production of H_2_O and CO_2_^12^.

Knowing that, in aerobic conditions, CO_2_ is the ultimate end product of the microbial respiration of organic substrates, the quantification of their level of biodegradation, once mixed or buried in active compost, can be directly assessed by measuring the total amount (cumulative) of CO_2_ respired over time^45^. To achieve this, several experimental procedures have been recognized by the scientific community^57^ and one of them has been adopted by ASTM^58^. ASTM D5338 is a detailed procedure that also describes the setup of the apparatus necessary for the rapid and reproducible determination of aerobic biodegradability under controlled composting conditions.

The results of our biodegradation tests on organic electronic materials carried out under mesophilic conditions at 25 °C revealed a mineralization rate of 4.1 ± 0.7% for Sepia Melanin, i.e., a rate 17× lower than 71.2 ± 0.2% for microcrystalline cellulose, the positive control (Fig. 2c and Table 1). Under thermophilic conditions (58 °C), the mineralization of Sepia Melanin reached a level 9× higher than that observed at 25 °C, still 2.6× lower than the 98 ± 6% mineralization level for cellulose (Fig. 3c and Table 1). Data show that the rate of melanin degradation increases with increasing incubation temperature (i.e., temperature coefficient *Q*_10_ = 1.95^59^), similarly to other biodegradation or bioremediation case studies. Further, they confirm the role of the thermophilic microbial community. Not only does the incubation temperature have an effect on the rate of melanin biodegradation because of its direct effect on the enzymes involved in the biodegradation but it also applies selective pressure on the microbiota of the compost that produces those enzymes. The two conditions used during the tests (25 °C and 58 °C) made it possible to select a community of mesophilic (optimal growth temperature is in the range 15–42 °C) or thermophilic (optimal growth temperature 42–70 °C) microorganisms^60,61^. Thermophilic microorganisms produce thermotolerant enzymes^62,63^ and exhibit higher respiration rates than mesophilic microorganisms, thus contributing to explain the significantly higher biodegradation rate observed at 58 °C. The fundamental difference between mesophilic and thermophilic organisms in terms of respiration rate at their respective optimal growth rate is well established in microbial ecology and it was noticeably confirmed by our observations of the blank compost incubated in those two temperature ranges (“Characterization of Blank Compost” in SI).

In order to explain the high degradation levels of cellulose (71% at 25 °C and 98% at 58 °C), it must be considered that the compost we used was from a facility dedicated to the biodegradation of lignocellulosic biomasses. On the other hand, the growth kinetics and the structure of the mesophilic microorganisms community compared to the thermophilic one as well as the intrinsic biodegradation-hindering factors of eumelanin (structural and chemical disorder, as will be discussed later) could explain its recalcitrance to biodegradation at 25 °C and its moderate biodegradation level at 58 °C (i.e., 4.1 and 37%, respectively). Further microbiological tests are needed, to assess the community structure of our compost. It is worth noting that the fungus *Aspergillus fumigatus* that biodegrades eumelanin at room temperature can tolerate environments of 50 °C and above^64^.

Although the biodegradation level under composting conditions (37% in 98 days) is significant, it does not meet the current industrial requirements for its labeling as biodegradable under composting conditions (ASTM D6400). As a matter of fact, even if we project the biodegradation level at the upper time limit suggested by ASTM D6400, 180 days, using the 0.26%/day mineralization rate observed during the incubation period corresponding to Δ*t*_35–98_, Sepia Melanin would reach 58%, still not fulfilling the 90% threshold of biodegradability in composting conditions.

Our results suggest that the intrinsic characteristics of eumelanin, its molecular and supramolecular structure^65^ as well as its hygroscopicity^21^, affect the extent of its biodegradation by the compost microbiota. It is well established that eumelanin structure, rather than being composed of linear homopolymeric chains (e.g., cellulose and starch), is based on the oligomeric DHI and DHICA monomers (Fig. 1)^65^. The complexity of eumelanin structure (chemical disorder) also stems from the fact that oligomers differ from each other in the number of units, types of units (DHI vs. DHICA), and sites of polymerization (similarly to lignin whose supramolecular structure emerges from the assembly of three monomeric crosslinked monolignols: p-coumaryl alcohol, coniferyl alcohol, and sinapyl alcohol)^66^.

Eumelanin oligomers organize in a complex supramolecular structure: the oligomeric sheets form protoparticles via π–π stacking. The protoparticles arrange in an onion-like structure, densify into spherical particles (about 10 nm-sized) and eventually undergo aggregation into larger spherical particles (ca. 100 nm-sized)^65^. Eumelanin particles feature heterogeneity in supramolecular structure and size. In other words, the chemical disorder of eumelanin is paralleled by structural (physical) disorder^22^.

The chemical and structural disorder may limit the biodegradability of eumelanin, since not all oligomeric planes are exposed to the depolymerizing enzymes secreted by the microbiome and diffused in the compost. The monomer-monomer bonds may undergo enzymatic degradation only from the “external” oligomers (i.e., on the surface) of eumelanin particles. This might explain the observed low rate of biodegradation. It has been suggested that the “opening” of the supramolecular structure, i.e., the de-stacking of the oligomers, is a necessary step for biopigment degradation^67^.

As Sepia Melanin is a heterogeneous substrate, we hypothesize that its biodegradation requires several types of extracellular enzymes to achieve complete biodegradation, via a mechanism very similar to the enzymatic oxidation of lignin, based on manganese peroxidase (Mn–P) and lignin peroxidase (Li–P)^54,68^. Moreover, a type of enzyme able to act on one type of eumelanin oligomer may have a different affinity towards different oligomers^33^, whereas a non-biodegraded oligomer will limit the exposure to extracellular enzymes of all the oligomers stacking beneath it.

Furthermore, eumelanin is known to be a hygroscopic material^21^. The extracted Sepia Melanin absorbs 17 wt% of H_2_O in 1 h at 90% relative humidity (Supplementary Fig. 2, Supplementary Table 1). We already reported that in 24 h Sepia Melanin can absorb a water quantity equal to its weight^21^. Knowing that the compost used contained a significant amount of water, equivalent to 50% of its initial mass, it is reasonable to assume that hydration of Sepia Melanin took place in the bioreactor, which could have contributed to weakening the high chemical and physical stability level of melanin, thereby allowing its biodegradation. Indoles are indeed known to be microbially degraded with the indole ring cleavage following a pathway that ends with the formation of fumarate and pyruvate^69^. In addition, water would solubilize the resulting monomers or the low molecular weight biodegradation products, which could then diffuse into the microbial cells to be metabolized^70^. Consequently, biopigment hygroscopicity favors biodegradation.

Seedling emergence and plant biomass thresholds established for the terrestrial phytotoxicity tests were attained and surpassed by Sepia Melanin (Table 2). Thus, our ecotoxicological results show that the “residues” of the partially mineralized pigment are not phytotoxic. Although L-dopa, the precursor of melanin, shows phytotoxic effects due to the formation of reactive oxygen species and/or free-radical species during melanogenesis^71^, others report that fumarates resulting from the biodegradation of indoles^69^ would not be phytotoxic^72^.

The apparent respiration activity of Cu–Pc blended with compost initially (Δ*t*_0–50_) falls within the range of the blank compost, but then, after 50 days, it becomes lower, which implies that there was no net CO_2_ production with respect to the blank compost and, consequently, no biodegradation (Fig. 4). This trend could be explained with a partial release of Cu ions by the synthetic molecule: such cations are able to inhibit microbial activity^73^. The molecular structure of Cu–Pc presents a planar aromatic macrocycle with the metal cation at its center^74^ (Fig. 1). Such a molecular structure is based on indoles, like that of eumelanin. However, in Cu–Pc, the four indole units do not include catechol groups and are not directly chemically bound to each other^74^ (Fig. 1). The absence of biodegradation of Cu–Pc could also be due to inhibition of microbial metabolism by the released copper cations and to resistance to enzymatic hydrolysis of the nitrogen-including bonds between the indole units^73^. The π–π stacking of the molecules of Cu–Pc and their insolubility in water may represent additional biodegradation-hindering factors^74^. Prior to this study, no phytotoxicity test had been conducted with Cu–Pc, although the phytotoxicity of several other phthalocyanines has been evaluated^75^. Following the biodegradation test, the “residual” Cu–Pc in the compost shows no phytotoxic effects (Table 2).

The apparent respiration rate of PPS blended with compost falls within the range of the blank compost over the entire incubation period (Δ*t*_0–98_) (Table 1 and Fig. 4). Consequently, no mineralization took place. The chemical bond between a sulfur atom and a benzene ring in a synthetic polymer appears to be resistant to the action of numerous extracellular hydrolytic enzymes present in the compost under thermophilic conditions (Fig. 1). PPS does not pass the thresholds for two phytotoxicity tests; it limits both the emergence of seedlings and the growth of plants. This points to the potential phytotoxicity of the polymer (Table 2).

We wish to emphasize that “bioresorbability” does not equate to “biodegradability”. The concept of bioresorbability applies to transformations in an aqueous physiological environment (mainly in vivo), while biodegradability refers to biotic processes catalyzed by enzymatic reactions within communities of microorganisms active in their environment or ecosystem^48,76^. For example, Bettinger et al. report that synthetic melanin implants were almost completely resorbed after 56 days of in vivo exposure^31^, which differs significantly from the experimental ecosystem we have used to observe the biodegradation of Sepia Melanin in the compost after 98 days at 58 °C. Although incomplete, our mineralization result (i.e., 37%) reveals an average specific degradation rate of 0.63 g melanin per day per kg dry weight compost.

Abundant and biodegradable organic (carbon-based) electronic materials and devices represent one of the viable ways of reducing the environmental footprint of the electronic sector. At present, no guidelines for testing the biodegradability of organic electronic materials and devices are available. Green organic electronics have access only to a test protocol specifically dedicated to the certification of compostable plastic materials. Our work is the first of its kind in the development of new guidelines and test protocols.

We found that eumelanin, an organic electronic bio-sourced material, attained a mineralization level of 37% after 97 days under composting conditions (at 58 °C); plant seedling and germination tests revealed that the residual material at the end-point of the composting test did not exhibit phytotoxic effects. We also tested the biodegradability in composting conditions of two non-bio-sourced (synthetic) organic electronic materials, namely copper (II) phthalocyanine, Cu–Pc, and poly(1–4)phenylene sulfide, PPS. The conjugated molecular structures of Cu–Pc and PPS feature C=C bonds (for Cu–Pc and PPS) and C=N (for Cu–Pc) bonds as well as the absence of oxygen atoms, characteristics that help explain the negligible mineralization levels observed. Finally, Cu–Pc inhibited the respiration of the microorganisms while PPS showed potential phytotoxicity. These differences suggest that bio-sourced materials constitute a viable option for the eco-design of organic electronic devices.

We wish to point out that CO_2_ monitoring is an “acceptable” analytic method for assessing the biodegradation of a given material buried in a substrate as complex as compost. Nevertheless, the method does not provide information about the material’s half-life and the identification of the microbes responsible for biodegradation. A series of tests including radio-respirometry, extraction of the residual test material, and in vitro tests using commercially available enzymes or isolated microbes are deemed necessary to properly describe the mechanism of biodegradation. Such tests are not part of ASTM D5338.

Further studies exploring other types of compost, e.g., from manure or garden waste, that feature different microbial communities, or other types of compost facilities, e.g., home composting for which different standards exist, are needed to identify the most suitable conditions for eumelanin’s biodegradability. In addition, to accelerate the degradation process inoculation and biostimulation are envisaged and can be adopted since microbial populations can evolve and derive their energy from an array of chemicals of different origins^77^. Work is in progress towards the evaluation of the biodegradability of organic electronic devices, beyond the constituent materials.

## Methods

### Chemicals

Three test materials were selected on the basis of their availability and properties: Sepia Melanin, copper(II) phthalocyanine (Cu–Pc), and polyphenylen sulfide (PPS). Sepia Melanin was extracted from the ink sac of a cuttlefish (*Sepia officinalis*) following established extraction and purification procedures^78^ (“Extraction of Sepia Melanin” in Supplementary Information (SI)). Four chemicals, purchased from Sigma-Aldrich Canada, were used as received: microcrystalline cellulose (20 μm powder), PPS (powder, average *M*_*n*_~10,000), Cu–Pc (β-form, dye content 90 wt%), and Ba(OH)_2_. Linear low-density PE was purchased from Exxon Mobil^TM^ (LL 6407.67). Extracted Sepia Melanin was characterized by IR, TGA, NAA, as well as elemental analyses of total carbon (CHN analysis) and TIC (“Characterization of Sepia Melanin” in SI: Supplementary Figs. 1 and 2 and Supplementary Tables 1 and 2).

### Compost characteristics

Compost from solid municipal wastes (Englobe Corp.) was used as the *substratum* in the biodegradation tests. However, beforehand, tests were performed to ensure the blank compost met the requirements of ASTM D5338: pH 7.3, ash content 51.4%, dry solids 46 ± 4 wt%, C/N ratio 24.3 with %C = 50% organic matter, and the specific respiration rate 10.5 ± 0.1 mg CO_2_ evolved per g of volatile solid per day (“Characterization of Blank Compost” in SI)^45^.

### Biodegradability test under mesophilic conditions (25 °C)

The aerobic biodegradability of Sepia Melanin, in powder form, under mesophilic conditions was tested over an incubation period of 97 days. The respiration activity of the compost microbiota was monitored by means of electrolytic respirometers^79^. This equipment continuously measured consumed O_2_, one data point every 30 min (Monitoring O_2_ Consumption Using “Electrolytic Respirometers” and “Biodegradability Test under Mesophilic Conditions” in SI). The test also required the biodegradable material microcrystalline cellulose as the positive control.

### Biodegradability test under thermophilic conditions (58 °C)

The biodegradability test under thermophilic conditions involved four ingredients in the form of fine powder: Sepia Melanin, Cu–Pc, PPS, and microcrystalline cellulose as the positive control, and one ingredient in the form of 3-mm nominal-size granules of low-density PE as the negative control. Under the test conditions, cellulose and PE are, respectively, biodegradable and non-biodegradable. Over an incubation period of 98 days, the aerobic respiration activity of the compost microbiota was monitored, measuring the CO_2_ evolved (one data point every 4 days). Other phenomena such as non-photosynthetic CO_2_ fixation and abiotic degradation of eumelanin were not significant (“Monitoring CO_2_ Evolution Using Wet Scrubbers” in SI)^80–82^. The lab-scale composting facility consisting of up to sixteen 6-L glass bioreactors was maintained at a constant temperature of 58 °C and in darkness at all times. To trap the respired CO_2_, a wet scrubber consisting of three 1-L bottles, each filled with 900 mL of 0.12 M Ba(OH)_2_ solution, was attached to each of the bioreactors. The strong base Ba(OH)_2_ reacted with the gaseous CO_2_ respired by the compost microbiota. The quantity of CO_2_ was obtained by titration of two 30-mL samples which were taken from each of the Ba(OH)_2_ traps (“Monitoring CO_2_ Evolution Using Wet Scrubbers” and “Biodegradability Test in Composting Conditions” in SI). The average obtained was used to calculate the subtotal of CO_2_ respired, which was trapped by the 900-mL Ba(OH)_2_ solution. This subtotal was added to the subtotals of the other two traps, to obtain the total respired CO_2_ since the last sampling.

### Statistics and mineralization computations

Biodegradability tests featured the 1:6 weight ratio of test material to dry compost in accordance with ASTM D5338^58^. Keeping this ratio fixed, the amount of compost by weight was changed in the two biodegradation tests. The tests, carried out using 1-L or 6-L bioreactors, were performed in duplicate or in triplicate, as described in “Biodegradability Test under Mesophilic and in Composting Conditions” in SI. The mean value and standard deviation of the O_2_ consumed and CO_2_ evolved were calculated from the data obtained for each of the materials tested under mesophilic and thermophilic conditions. Details of the statistical analysis are provided in “Statistical analysis” in SI.

The biochemical reaction associated with the aerobic respiration of the compost microbiota can be simplified as:

1 $${{\rm{C}}}_{{\rm{TEST}}{\rm{MATERIAL}}}{+{\rm{O}}}_{2{\rm{CONSUMED}}}- > {{\rm{CO}}}_{2{\rm{RESPIRED}}}+{{\rm{H}}}_{2}{\rm{O}}+{{\rm{C}}}_{{\rm{RESIDUAL}}{\rm{TEST}}{\rm{MATERIAL}}}+{{\rm{C}}}_{{\rm{BIOMASS}}}$$

where C_*TEST MATERIAL*_ is the carbon of the test material at the beginning of the test; C_*RESIDUAL TEST MATERIAL*_ is the carbon of the test material that is not biodegraded; and C_*BIOMASS*_ is the carbon assimilated and transformed into the biomass (compost microbiota) participating in the respiration process.

Consequently, the mineralization percentage is determined as follows^45^:

2 $${\mathrm{Mineralization}}( \% )=\frac{{\mathrm{C}}{{\mathrm{O}}}_{2}({\mathrm{test}}\,{\mathrm{material}})-{\mathrm{C}}{{\mathrm{O}}}_{2}({\mathrm{blank}}\,{\mathrm{compost}})}{{\mathrm{C}}{{\mathrm{O}}}_{2}({\mathrm{total}})}$$

where CO_2_ (test material) is the measured mass of CO_2_ evolved from the bioreactor containing the test material blended with or buried in the compost, CO_2_ (blank compost) is the average measured mass of CO_2_ evolved from the bioreactors containing blank compost, and CO_2_ (total) corresponds to the total theoretical mass of CO_2_ expected if all the test material were completely respired by the compost microbiota, according to the following equation:

3 $${\mathrm{C}}{{\mathrm{O}}}_{2}({\mathrm{total}})({\mathrm{g}})={{\mathrm{C}}}_{ \% }* {{m}}* \frac{44.01({\mathrm{C}}{{\mathrm{O}}}_{2}\,{\mathrm{molecular}}\,{\mathrm{mass}})}{12.01({\mathrm{C}}\,{\mathrm{atomic}}\,{\mathrm{mass}})}$$

where C_%_ is the mass percentage of carbon in the test material and *m* is the mass of the test material.

With regard to the biodegradation test under mesophilic conditions, the recorded data of O_2_ consumed over time were converted (computed) to CO_2_ respired (the numerator of Eq. (2)) following Eq. (1). To solve these two equations, we have used a respiratory quotient (RQ), i.e., CO_2_ respired over O_2_ consumed, of 1.0 mol/mol^83^. The standard deviation of the mineralization was computed as per ASTM D5338 (“Mineralization” in SI).

### Phytotoxicity test

Immediately after the endpoint of the biodegradability test in thermophilic conditions, a germination test and a growth test were conducted to evaluate the potential terrestrial phytotoxicity of the residual test material and, if any, of their biodegradation products. Such tests are recommended in ASTM D6400. The phytotoxicity test procedure was adapted from OECD Guideline 208^84^. Samples were mixed with wet sandy soil, then incubated for 19 days at 25 °C. Three parameters were monitored: the seedling emergence of 20 ryegrass seeds, the absence of visible phytotoxic effects (e.g., chlorosis, necrosis, leaf, and stem deformation), and the biomass production (wet weight of the resulting plants) after 19 days (“Phytotoxicity Test” in SI). ASTM D6400 suggests using two plant species for the phytotoxicity test: consequently, our test has to be considered only as a first indication of the terrestrial phytotoxicity of the materials. To reinforce the significance of the phytotoxicity test, the rate of respiration of the compost microbiota (as observed during the biodegradability test at 58 °C) was used as a second bioindicator of potentially toxic effects for the terrestrial trophic level^85^.

## Supplementary information

Supplementary Information

## Data Availability

The datasets generated during and/or analyzed during the current study are available from the corresponding author on reasonable request.

## References

[1] Balde, C. P., Forti, V., Gray, V., Kuehr, R. & Stegmann, P. *The Global E-waste Monitor 2017: Quantities, Flows and Resources*. (United Nations University, International Telecommunication Union, and International Solid Waste Association, 2017).

[2] Baumgartner, M. et al. Emerging “green” materials and technologies for electronics. in *Green Materials for Electronics* (eds. Irimia-Vladu, M., Glowacki, E. D., Sariciftci, N. S. & Bauer, S.) 1–53 (Wiley-VCH Verlag GmbH & Co. KGaA, 2017). 10.1002/9783527692958.ch1

[3] Zvezdin A, Di Mauro E, Rho D, Santato C, Khalil M (2020). En route toward sustainable organic electronics. MRS Energy Sustain.

[4] Feig VR, Tran H, Bao Z (2018). Biodegradable polymeric materials in degradable electronic devices. ACS Cent. Sci..

[5] Lei, T. et al. Biocompatible and totally disintegrable semiconducting polymer for ultrathin and ultralightweight transient electronics. *Proc. Natl. Acad. Sci*. *USA* 201701478 (2017). 10.1073/PNAS.1701478114 10.1073/pnas.1701478114 PMC5441761 28461459

[6] Heeger AJ (2001). Semiconducting and metallic polymers: the fourth generation of polymeric materials (nobel lecture). Angew. Chem. Int. Ed..

[7] Tietze ML (2018). Elementary steps in electrical doping of organic semiconductors. Nat. Commun..

[8] Park S (2018). Self-powered ultra-flexible electronics via nano-grating-patterned organic photovoltaics. Nature.

[9] Root SE, Savagatrup S, Printz AD, Rodriquez D, Lipomi DJ (2017). Mechanical properties of organic semiconductors for stretchable, highly flexible, and mechanically robust electronics. Chem. Rev..

[10] Ghasemi Ghodrat, A., Tabatabaei, M., Aghbashlo, M. & Mussatto, S. I. Waste management strategies; the state of the art. in *Biogas: Fundamentals, Process, and Operation* (eds. Tabatabaei, M. & Ghanavati, H.) 1–33 (Springer International Publishing, 2018). 10.1007/978-3-319-77335-3_1

[11] Falkiewicz-Dulik, M., Janda, K. & Wypych, G. Introduction. in *Handbook of Material Biodegradation, Biodeterioration, and Biostablization* 1–6 (Elsevier, 2015). 10.1016/C2014-0-01354-8

[12] van der Zee, M. Methods for evaluating the biodegradability of environmentally degradable polymers. in *Handbook of Biodegradable Polymers* 1–28 (Smithers Rapra Technology Ltd, 2014).

[13] Specifications for compostable plastics CAN/BNQ 0017-088/2010 (ISO 17088: 2008, MOD). (2010).

[14] ASTM D6400-12, Standard specification for labeling of plastics designed to be aerobically composted in municipal or industrial facilities. *ASTM Int.* **1**, 1–3 (2012).

[15] EN 13432:2000. Packaging—requirements for packaging recoverable through composting and biodegradation—test scheme and evaluation criteria for the final acceptance of packaging.

[16] EN 14995. Plastics—evaluation of compostability—test scheme and specifications.

[17] Castro-Aguirre E, Auras R, Selke S, Rubino M, Marsh T (2017). Insights on the aerobic biodegradation of polymers by analysis of evolved carbon dioxide in simulated composting conditions. Polym. Degrad. Stab..

[18] Jablonski NG, Chaplin G (2010). Human skin pigmentation as an adaptation to UV radiation. Proc. Natl Acad. Sci. USA.

[19] d’Ischia M (2013). Melanins and melanogenesis: methods, standards, protocols. Pigment Cell Melanoma Res.

[20] Prota G (1995). The chemistry of melanins and melanogenesis. Fortschr. der Chem. Org. Naturst..

[21] Albano LG (2016). Novel insights on the physicochemical properties of eumelanins and their DMSO derivatives. Polym. Int..

[22] Meredith P, Sarna T (2006). The physical and chemical properties of eumelanin. Pigment Cell Res..

[23] Shanmuganathan K, Cho JH, Iyer P, Baranowitz S, Ellison CJ (2011). Thermooxidative stabilization of polymers using natural and synthetic melanins. Macromolecules.

[24] Xu R (2017). An electrochemical study of natural and chemically controlled eumelanin. APL Mater..

[25] Wünsche J (2015). Protonic and electronic transport in hydrated thin films of the pigment eumelanin. Chem. Mater..

[26] Mostert AB, Rienecker SB, Noble C, Hanson GR, Meredith P (2018). The photoreactive free radical in eumelanin. Sci. Adv..

[27] Reali M (2020). Electronic transport in the biopigment sepia melanin. ACS Appl. Bio Mater..

[28] Kumar P (2016). Melanin-based flexible supercapacitors. J. Mater. Chem. C.

[29] Kim YJ, Wu W, Chun S-E, Whitacre JF, Bettinger CJ (2013). Biologically derived melanin electrodes in aqueous sodium-ion energy storage devices. Proc. Natl Acad. Sci. USA.

[30] Sheliakina M, Mostert AB, Meredith P (2018). An all-solid-state biocompatible ion-to-electron transducer for bioelectronics. Mater. Horiz..

[31] Bettinger CJ, Bruggeman JP, Misra A, Borenstein JT, Langer R (2009). Biocompatibility of biodegradable semiconducting melanin films for nerve tissue engineering. Biomaterials.

[32] Ribera J (2018). Scalable biosynthesis of melanin by the basidiomycete armillaria cepistipes. J. Agric. Food Chem..

[33] Blois, M. S. Random polymers as a matrix for chemical evolution—example of melanin. in *The Origins of Prebiological Systems and of Their Molecular Matrices* (ed. Fox, S. W.) 19–33 (Academic Press, Inc., 1965).

[34] Kuo M, Alexander M (1967). Inhibition of the lysis of fungi by melanins. J. Bacteriol..

[35] Wilson AS, Dodson HI, Janaway RC, Pollard AM, Tobin DJ (2007). Selective biodegradation in hair shafts derived from archaeological, forensic and experimental contexts. Br. J. Dermatol..

[36] Lindgren J (2012). Molecular preservation of the pigment melanin in fossil melanosomes. Nat. Commun..

[37] Kim BS, Blaghen M, Hong H-S, Lee K-M (2016). Purification and characterization of a melanin biodegradation enzyme from Geotrichum sp. Int. J. Cosmet. Sci..

[38] Luther JP, Lipke H (1980). Degradation of melanin by Aspergillus fumigatus. Appl. Environ. Microbiol.

[39] Mohorčič M (2007). Production of melanin bleaching enzyme of fungal origin and its application in cosmetics. Biotechnol. Bioprocess Eng..

[40] Malliaras G, Friend R (2005). An organic electronics primer. Phys. Today.

[41] ELEY DD (1948). Phthalocyanines as semiconductors. Nature.

[42] Bao Z, Lovinger AJ, Dodabalapur A (1996). Organic field-effect transistors with high mobility based on copper phthalocyanine. Appl. Phys. Lett..

[43] Kinoshita, Y., Hasobe, T. & Murata, H. Control of open-circuit voltage in organic photovoltaic cells by inserting an ultrathin metal-phthalocyanine layer. *Appl. Phys. Lett*. **91**, 083518 (2007).

[44] Rahate AS, Nemade KR, Waghuley SA (2013). Polyphenylene sulfide (PPS): state of the art and applications. Rev. Chem. Eng..

[45] ASTM D5338-2015. *Standard Test Method for Determining Aerobic Biodegradation of Plastic Materials Under Controlled Composting Conditions*. (2015). 10.1520/D5338-15.2

[46] Araújo M (2014). Natural melanin: a potential pH-responsive drug release device. Int. J. Pharmaceut..

[47] Williams, D. F. B. in *The Williams Dictionary of Biomaterials* 33–54 (Liverpool University Press, 2012). 10.5949/UPO9781846314438.006

[48] Liu Y, Zheng Y, Hayes B (2017). Degradable, absorbable or resorbable—what is the best grammatical modifier for an implant that is eventually absorbed by the body?. Sci. China Mater..

[49] Chang J-K (2018). Biodegradable electronic systems in 3D, heterogeneously integrated formats. Adv. Mater..

[50] Pal RK, Kundu SC, Yadavalli VK (2018). Fabrication of flexible, fully organic, degradable energy storage devices using silk proteins. ACS Appl. Mater. Interfaces.

[51] Hu L, Dai J, Carter M, Wang Z, Fu KK (2016). Transient electronics: materials and devices. Chem. Mater..

[52] Mason, I. G. A study of power, kinetics, and modelling in the composting process. (University of Canterbury, 2007). 10.26021/2348

[53] Paice MG (1984). Two forms of endoglucanase from the basidiomycete Schizophyllum commune and their relationship to other β-1,4-glycoside hydrolases. Nat. Biotechnol..

[54] Kiran S (2019). Lignin degrading system of phanerochaete chrysosporium and its exploitation for degradation of synthetic dyes wastewater. Pol. J. Environ. Stud..

[55] Tuomela M (2000). Biodegradation of lignin in a compost environment: a review. Bioresour. Technol..

[56] Rho D, Desrochers M, Jurasek L, Driguez H, Defaye J (1982). Induction of cellulose in Schizophyllum commune: thiocellobiose as a new inducer. J. Bacteriol..

[57] Gómez RB, Lima FV, Ferrer AS (2006). The use of respiration indices in the composting process: a review. Waste Manag. Res..

[58] ASTM International. ASTM D5338-15: Standard test method for determining aerobic biodegradation of plastic materials under controlled composting conditions, conditions, incorporating thermophilic temperatures. *ASTM Stand*. (2015). 10.1520/D5338-15.2

[59] Kvicala, J. L. The Effect of Temperature on the Rate and Extent of Crude Oil Biodegradation in a Soil Slurry. (University of Calgary, 2001). 10.11575/PRISM/11471

[60] Pietikäinen J, Pettersson M, Bååth E (2005). Comparison of temperature effects on soil respiration and bacterial and fungal growth rates. FEMS Microbiol. Ecol..

[61] Tang Z, Sun X, Luo Z, He N, Sun OJ (2018). Effects of temperature, soil substrate, and microbial community on carbon mineralization across three climatically contrasting forest sites. Ecol. Evol..

[62] Tang J-C, Shibata A, Zhou Q, Katayama A (2007). Effect of temperature on reaction rate and microbial community in composting of cattle manure with rice straw. J. Biosci. Bioeng..

[63] Insam, H. & de Bertoldi, M. Chapter 3 Microbiology of the composting process. in *Compost Science and Technology* (eds. Diaz, L., de Bertoldi, M., Bidlingmaier, W. & Golueke, C.) **154**, 25–48 (Elsevier Waste Management Series, 2007).

[64] Bhabhra R, Askew DS (2005). Thermotolerance and virulence of Aspergillus fumigatus: role of the fungal nucleolus. Med. Mycol..

[65] Büngeler, A., Hämisch, B., Huber, K., Bremser, W. & Strube, O. I. Insight into the final step of the supramolecular buildup of eumelanin. *Langmuir* **33**, (2017). 10.1021/acs.langmuir.7b01634 28639791

[66] Tran ML, Powell BJ, Meredith P (2006). Chemical and structural disorder in eumelanins: a possible explanation for broadband absorbance. Biophys. J..

[67] Borovanský J, Elleder M (2003). Melanosome degradation: fact or fiction. Pigment Cell Res.

[68] Barr DP, Aust SD (1994). Mechanisms white rot fungi use to degrade pollutants. Environ. Sci. Technol..

[69] Claus G, Kutzner HJ (1983). Degradation of indole by Alcaligenes spec. Syst. Appl. Microbiol..

[70] Grima S, Bellon-Maurel V, Feuilloley P, Silvestre F (2000). Aerobic biodegradation of polymers in solid-state conditions: a review of environmental and physicochemical parameter settings in laboratory simulations. J. Polym. Environ..

[71] Hachinohe M, Matsumoto H (2007). Mechanism of selective phytotoxicity of L-3,4-dihydroxyphenylalanine (L-dopa) in barnyardglass and lettuce. J. Chem. Ecol..

[72] Jha P (2013). Biodegradation of phenol using hairy roots of Helianthus annuus L. Int. Biodeterior. Biodegrad..

[73] Said WA, Lewis DL, Ecology F (1991). Quantitative assessment of the effects of metals on microbial degradation of organic. Chemicals.

[74] Claessens CG, Hahn U, Torres T (2008). Phthalocyanines: from outstanding electronic properties to emerging applications. Chem. Rec..

[75] Jančula D, MarŠálek B (2012). The toxicity of phthalocyanines to the aquatic plant Lemna minor (duckweed)—testing of 31 compounds. Chemosphere.

[76] Pradhan S, Brooks AK, Yadavalli VK (2020). Nature-derived materials for the fabrication of functional biodevices. Mater. Today Bio.

[77] Austin HP (2018). Characterization and engineering of a plastic-degrading aromatic polyesterase. Proc. Natl Acad. Sci. USA.

[78] Magarelli M, Passamonti P, Renieri C (2010). Purification, characterization and analysis of sepia melanin from commercial sepia ink (Sepia Officinalis) Purificación, caracterización y análisis de la melanina de sepia a partir de la tinta de sepia (Sepia Officinalis) Resumen. Rev. CES Med. Vet. Zootec..

[79] Fradette S, Rho D, Samson R, LeDuy A (1994). Microcalorimetry as a diagnostic and analytical tool for the assessment of biodegradation of 2,4-D in a liquid medium and in soil. Appl. Microbiol. Biotechnol..

[80] Miltner A, Richnow H-H, Kopinke F-D, Kästner M (2005). Incorporation of carbon originating from CO_2_ into different compounds of soil microbial biomass and soil organic matter. Isotopes Environ. Health Stud..

[81] Miltner A (2005). Non-phototrophic CO_2_ fixation by soil microorganisms. Plant Soil.

[82] Yuan H, Ge T, Chen C, O’Donnell AG, Wu J (2012). Significant role for microbial autotrophy in the sequestration of soil carbon. Appl. Environ. Microbiol..

[83] Rho D, André G (1991). Growth and stoichiometry of a Catharanthus roseus cell suspension culture grown under nitrogen-limiting conditions. Biotechnol. Bioeng..

[84] Test No. 208: *Terrestrial Plant Test: Seedling Emergence and Seedling Growth Test*. (OECD, 2006). 10.1787/9789264070066-en

[85] Gong P (2000). An in situ respirometric technique to measure pollution-induced microbial community tolerance in soils contaminated with 2,4,6-trinitrotoluene. Ecotoxicol. Environ. Saf..

