# Supplementary material for: Biodegradation of bio-sourced and synthetic organic electronic materials towards green organic electronics
Source: Nat Commun. 2021 May 26;12:3167. doi: 10.1038/s41467-021-23227-4 (PMC8154894; doi:10.1038/s41467-021-23227-4)
Supplement: Supplementary file 1 — Supplementary Information [file 41467_2021_23227_MOESM1_ESM.pdf]

Supplementary Information, Biodegradation of Bio-Sourced and Synthetic Organic Electronic Materials Towards Green Organic Electronics

Eduardo Di Mauro, Denis Rho, Clara Santato

Extraction of Sepia Melanin

Sepia Melanin was extracted from cuttlefish ink (Seafoods Boston, Product of Spain), bought in a Montréal fish market, according to a procedure adapted from existing protocols[1,2]. Eleven centrifugations and wash-suspensions were carried out, sequentially, on the cuttlefish ink with different solvents: hydrochloric acid, ethanol, ethyl acetate (to remove carbohydrates, proteins and lipids), and deionized water (DI). First, the commercial cuttlefish ink (2 kg) was vigorously shaken, then poured in a HCl solution (2 M) into a dark recipient; the cuttlefish ink to acid ratio was 50 g to 100 mL. The slurry was mixed by magnetic stirring for 30 min. and kept for 24 h at 10 °C. Subsequently, the solids were separated from the supernatant by centrifugation (10000 rpm at 5 °C for 15 min.). The solids were washed-suspended: 3x with HCl solution (0.5 M), 1x with deionized water; 1x with phosphate buffer (monobasic sodium phosphate (200 mM, 95 mL) and 1x with dibasic sodium phosphate (200 mM, 405 mL) per 500 mL of DI water), 2x with DI water, 1x with ethanol, 1x with ethyl acetate, 1x with DI water. Prior to each centrifugation, the suspension was vigorously shaken using a Vortex mixer. The last step entailed 24-hour freeze-drying (lyophilization) to obtain solvent-free melanin powder. In the end, approximately 200 g of black powder was obtained, thereafter referred to as “Sepia Melanin extracted” (SM-E); and the extraction yield was approximately 10% by wt.

Ethyl acetate (99.8%) and ethanol (95%) were purchased from Sigma-Aldrich (Canada). Hydrochloric acid solution (12 M) was purchased from EMD Millipore (Canada). Commercial Sepia Melanin (SM-C) was purchased from Sigma Aldrich (product number M2649) and used as a reference material.

Characterization of Sepia Melanin

The extracted Sepia Melanin (SM-E) was characterized by means of infrared spectroscopy (IR), thermo-gravimetric analysis (TGA), neutron activation analysis (NAA), and elemental analyses of total carbon (CHN) and total inorganic carbon (TIC).

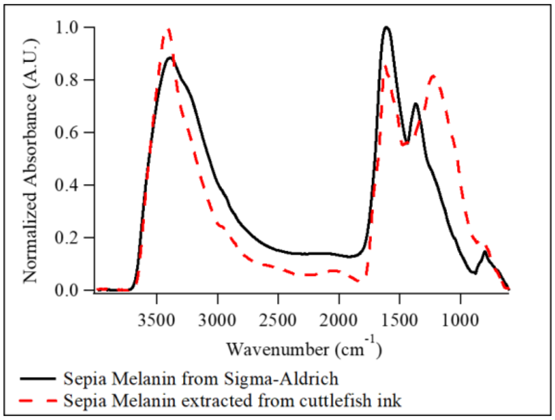

Supplementary Figure 1: IR spectra of commercial Sepia Melanin (—) and extracted Sepia Melanin (- - -).

The IR spectrum of the SM-E (Supplementary Figure 1) presents five peaks at: 3400 cm^{-1} (N-H stretching of the pyrrole ring), 2924 cm^{-1} (C-H stretching), 1600 cm^{-1} (C=C aromatic ring vibration/[NH_2] deformation), 1370 cm^{-1} (indole ring vibration/CNC stretching) and a broad band around 1220 cm^{-1} (C-H in-plane deformation, C-O stretching and O-H in-plane deformation in the carboxylic group COOH)[3].

Both weakly and strongly bound water can be present in Sepia Melanin after extraction[4,5]. Consequently, the dry solids (residual mass after heating the material at 105 °C until constant mass is reached) were quantified by means of a TGA. This measurement entailed a temperature ramp of 10 °C/min up to 105 °C, then an isothermal step at 105 °C, until the derivative (DTG) was less than 0.01 %/min in Ar atmosphere. The dry solids of SM-E amounted to 83.1 ± 0.3% wt. (n = 5), while for SM-C it was 81.9 ± 0.4% wt. (n = 2).

Thermogravimetric analyses provide insight into the thermal decomposition of a material. In particular, the minima of the DTG represent the completion of a thermogravimetric phenomenon and its maxima represent the maximum rate at which a thermogravimetric phenomenon takes place[5]. The TGAs of the two powders were carried out in inert atmosphere, ramp of 10 °C/min between 25° – 500 °C. The thermal decomposition of SM-E and SM-C featured the same trend (Supplementary Figure 2a). The DTG has in both cases a first maximum (approximately 45 °C) followed by a minimum (approximately 140 °C), attributable to the maximum rate and to the end of the loss of weakly bound water, respectively. A step involving the decarboxylation of the DHICA units and opening of the supramolecular structure (Supplementary Figure 2 and Supplementary Table 1)[5] follows. At 500 °C, the mass loss is 60% wt. in both cases. The hygroscopicity was studied comparing the TGAs at two relative humidity (RH) levels, 25% and 90%. The mass loss at the first DTG minimum gives an approximate but comparable estimation of the difference in the amount of weekly bound water absorbed at a certain RH level[5]. The thermogravimetric results suggest that SM-C retains more weakly bound water in its supramolecular structure than SM-E, since the observed water loss from SM-C is 5-6% more by weight (Supplementary Table 1).

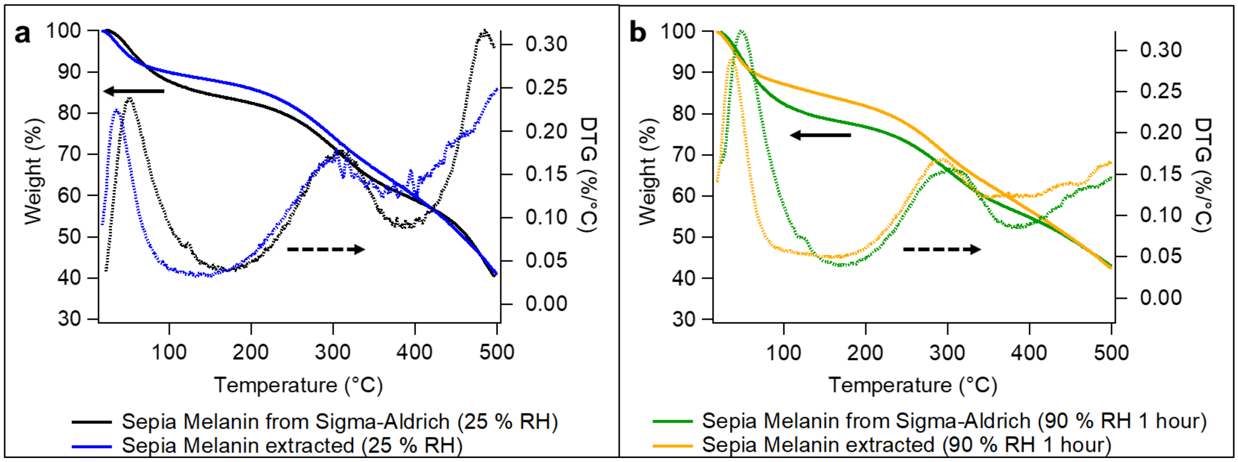

Supplementary Figure 2. Thermogravimetric analysis (TGA) of SM-E and SM-C (a) without any hydration treatment and (b) after 1 hour at 90% RH in inert N_2 and Ar atmosphere, respectively.

Supplementary Table 1. First minimum of the derivative of the TGA (DTG), attributable to the end of water loss, and the amount of weakly-bound water lost for Sepia Melanin from Sigma-Aldrich and Sepia Melanin extracted at 25% RH and 90% RH.

Eumelanin type	Relative Humidity (%)	First DTG minimum (°C)	Weight loss at first DTG minimum (%)
Sepia Melanin Extracted (SM-E)	25	138 ± 7	11.6 ± 0.4
	90	154 ± 12	16.6 ± 0.6
Sepia Melanin Commercial (SM-C)	25	169 ± 5	16.1 ± 0.3
	90	170 ± 1	22.2 ± 0.5

The composition of the inorganic matter was evaluated by means of neutron activation analysis (NAA), to determine what cations and anions are present after extraction (Supplementary Table *2*).

Supplementary Table 2. Analytical composition of Sepia Melanin (SM-E) by means of NAA.

Element	Value (ppm)	Element	Value (ppm)
Na	2685 ± 108	Cu	< 30
Ca	926 ± 201	Zn	< 20
Cl	919 ± 70	Cr	15 ± 1
Fe	479 ± 90	Co	7.36 ± 0.40
Mg	388 ± 80	Ag	< 7
K	< 170	Mn	6.99 ± 1.42
F	< 80	I	5.94 ± 0.95
Ni	< 30	Au	< 0.002

Monitoring O_2 Consumption Using Electrolytic Respirometers

Electrolytic respirometers (designed by Young *et al.*[6]) were used to follow the O_2 consumption (Model BI-2000, Bioscience, Inc.) under mesophilic conditions. In this experimental set-up, the compost is used in a closed environment (1.2-L bioreactor), in which the respired CO_2 is continuously trapped by a KOH solution (5 mL, 50% wt.), "suspended" within the bioreactor's headspace, while the respired O_2 is continuously replenished by the electrolytic cell.

Briefly, the opening of each glass bioreactor is occupied by the above-mentioned CO_2 trap, which is closed by a two-compartment electrolytic cell containing 25 mL of 1 N H_2SO_4 and a three-electrode cap (i.e. anode, cathode, and switch level). The compartment containing the anode is "exposed" to the pressure of the bioreactor's headspace and the other compartment containing the cathode is "exposed" to the pressure of the atmosphere. Noticeably, as the microorganisms present in the compost respire, oxygen (O_2) in the bioreactor's headspace is being used, which causes a pressure drop; with respect to the external pressure it corresponds to ΔP.

This ΔP activates the electrolytic cell by means of a switch electrode, i.e. water electrolysis. Consequently, at the cathode H_2 is produced and vented to the atmosphere, whereas at the anode O_2 is produced in the bioreactor's headspace until ΔP = 0. This mode of operation allows a discrete measurement of the O_2 consumed over time. Using Faraday's law, from the power provided to the electrolytic cell, the moles of O_2 produced can be computed. Further details of the working principle of the electrolytic respirometers can be found in reference[7]. The software BI2000 (modified *ad hoc* for the National Research Council Canada) was used to collect the data.

Monitoring CO_2 Evolution Using Wet Scrubbers

To monitor the microbial respiration of the tested materials, the CO_2 evolved under thermophilic conditions was monitored using an indirect method, cumulative measurement respirometry. All CO_2 produced, regardless of origin, was captured. Throughout the entire duration of the experiment, by means of an air compressor, 50 mL per minute of humidified air was "pushed" into each bioreactor and circulated within the compost to bring O_2 to the aerobic microorganisms and to release respired CO_2. Three traps filled with a $Ba(OH)_2$ solution were placed in a series after each bioreactor. As air came into the bioreactor, the same volume of air came out, not to the atmosphere but to a wet scrubber unit (i.e. a series of three 1-L bottles each one filled with 900 mL of 0.12 M $Ba(OH)_2$ solution), to precipitate all CO_2 respired.
The solution is able to capture CO_2 as the reaction (S1) takes place:

$$Ba(OH)_2 + CO_2(g) \rightarrow BaCO_3 + H_2O \qquad \text{(S1)}$$

At regular intervals, liquid samples taken from the 72 scrubbers [i.e. 8 bioreactors x 3 scrubbers/bioreactor x 3 (triplicate)] were titrated to calculate respired CO_2. During the titration, the remaining $Ba(OH)_2$ that has not reacted with CO_2 is neutralized with HCl 0.1 M:

$$Ba(OH)_2 + 2HCl \rightarrow BaCl_2 + 2H_2O \quad \text{(S2)}$$

From the molar ratios of equation (S2), at a certain incubation time *t*, it can be inferred that:

$$\text{moles } of\ CO_2\ trapped\ (t) = \text{moles of Ba}(OH)_2\ (t_0)\ - moles\ HCl\ (t)/2 \quad \text{(S3)}$$

Once a week, all scrubbers were replenished with freshly prepared $Ba(OH)_2$ solution to ensure full efficiency. The bioreactors (two replicates) containing the active compost were run specifically to monitor the background respiration level: essentially, [CO_2 production] - [CO_2 assimilation] = actual or observed CO_2 production. The background respiration was then subtracted from the material's respiration data to obtain the net respiration level for each test material. Consequently, this procedure, based on the equation [test] – [background] = net [CO_2 production], allowed us to calculate the biodegradation level based on its carbon (C) content.

Non-photosynthetic CO_2 fixation pathways are rarely important (marginal) in a nutrient-rich (high C/N ratio) ecosystem such as compost[8–10].

Characterization of Blank Compost

The compost, organic fraction of municipal solid waste, was kindly provided by the company *GSI Environnement*, subsidiary of Englobe (Québec, Canada). Its characteristics satisfied the requirements of ASTM D5338:

- sieved with a 10-mm mesh,
- ash content 51.4% over dry weight,
- pH 7.3,
- dry solids 46 ± 4 % over wet weight,
- C/N ratio 24.3 with %C = 50% organic matter over dry weight, and
- water content 50% over wet weight.

The figure of merit that indicates respiration activity of the compost falls within the acceptable range (i.e. fresh compost) is represented by the specific respiration rate, i.e. CO_2 evolved per g of volatile solids per day, mg CO_2/(g volatile solids ● d). Consequently, the specific respiration rate of the compost was measured both under mesophilic (25 °C) and thermophilic (58 °C) conditions with *ad hoc* tests, before the main tests that involved the materials of interest.

Under thermophilic conditions, the specific respiration rate was assessed deploying 6 bioreactors filled with 1 kg of blank wet compost and incubated for 11 days at 58 °C. The CO_2 evolved was followed using wet scrubbers (working mechanism in "Monitoring CO_2 Evolution Using Wet Scrubbers"). The result was 10.5 ± 0.1 mg CO_2/(g ● d), and, consequently, fell within the acceptable range set by ASTM D5338, i.e. 5 - 15 mg CO_2 evolved per g volatile solids per day, in the first 10 days.

Under mesophilic conditions, the specific respiration rate was measured deploying 7 bioreactors of 1.2 L filled with 100 g of blank wet compost and incubated for 7 days at 25 °C. As described above, the O_2

consumed was monitored by means of seven electrolytic respirometers. Then, CO_2 evolved was computed using the O_2 consumed data, assuming the respiratory quotient CO_2 respired / O_2 consumed to be 1.0 mol/mol[11] (working mechanism in "Monitoring O_2 Consumption using Electrolytic Respirometers"). Therefore, the average respiration rate was 1.4 ± 0.1 mg CO_2/(g ● d). This respiration rate, 8x lower than the rate measured under thermophilic conditions, is in agreement with the difference in the respiration rate between the two temperature ranges already reported in the literature[12].

Biodegradability Test Under Mesophilic Conditions

For the biodegradability test under mesophilic conditions (25 °C), bioreactors of 1.2 L were filled with 100 g of wet compost. A total of 7 bioreactors were used: two of plain compost (blank), two with cellulose blended with compost (positive control) and three with Sepia Melanin blended with compost. The weight ratio of material to dry compost was 1:6, as for the biodegradability test in composting conditions (ASTM D5338). Electrolytic respirometers (designed by Young *et al.*[6], model BI-2000, Bioscience Inc.) were used to follow the O_2 consumption (working mechanism in "Monitoring O_2 Consumption using Electrolytic Respirometers"). The CO_2 evolved was computed using the O_2 consumed data, assuming the respiratory quotient CO_2 respired / O_2 consumed to be 1.0 mol/mol[11].

Biodegradability Test in Composting Conditions

The standards ASTM D6400 and ASTM D5338-2015 define the terminology and the procedure to assess aerobic biodegradability of a plastic material in composting conditions[13,14].

In accordance with ASTM D5338, the respired CO_2 was monitored for 98 days. The Sepia Melanin blended with compost and the blank compost were tested using duplicates. The Cu-Pc, PPS, microcrystalline cellulose (positive control) and polyethylene (PE, negative control) blended or buried in compost were tested using triplicates. In each of the 16x 6-L bioreactors, 250 g of wet compost was present; and the materials were added to the compost in a dry weight ratio of 1:6. All bioreactors were kept in darkness at 58 °C in a controlled temperature chamber (Caron Stability Chamber) and continuously fed with air (flow of 32 ± 5 mL/min). The air passed through a stainless-steel aerator installed at the bottom of a 50-cm-high water column to be humidified before reaching the bioreactors.
Starting on day 47, the 16 bioreactors were opened once a week. Each of them was weighed and the difference between this mass and their respective initial mass was attributed to water loss. Consequently, DI water was added to keep the dry weight at or slightly below 50% wt. On average, the DI water added weekly was approximately 30 mL. While adding water to the compost, it was carefully mixed by hand. Then, each of them was reconnected to the bioreactors' air inlet and outlet.

The respired CO_2 was determined using an indirect measurement method, i.e. cumulative measurement respirometry (working mechanism detailed in the paragraph "Monitoring CO2 Evolution using Wet

Scrubbers"). Three traps filled with $Ba(OH)_2$ solution (1.8 L, 0.12 M) were installed in series at the outlet of each bioreactor. The air flowing through each of the bioreactors and their three traps was checked at least once a day. Regularly, i.e. on average, once every 4 days over 97 days, 2 liquid samples (30 mL) were withdrawn from each trap and titrated using HCl 0.1 M (certified, Fisher Scientific) with an automatic titrator (Metrohm, Switzerland, model 855). The titrator was interfaced with a Tiamo® Software version 2.5 and a pump model 772 was used to empty measured samples. The automatic titrator was also equipped with a 34-sample holder. For each sample, the average of two titrations was considered. Titrations were carried out also on the $Ba(OH)_2$ pristine solutions (approximately 0.12 M) that filled up the $Ba(OH)_2$ traps to measure their exact initial molarity, to compute the $\text{moles of Ba}(OH)_2\ (t = 0)$ of equation (S3).

The cumulative CO_2 evolved from the blank compost and from PE buried in compost is shown in Supplementary Figure 3. Throughout the whole incubation period, these two samples, the control compost and the PE buried in compost, featured equivalent apparent respiration rates falling within the same range, i.e. 457 ± 24 mg CO_2 / d and 488 ± 45 mg CO_2 / d in $\Delta t_{0\text{-}98}$, respectively.

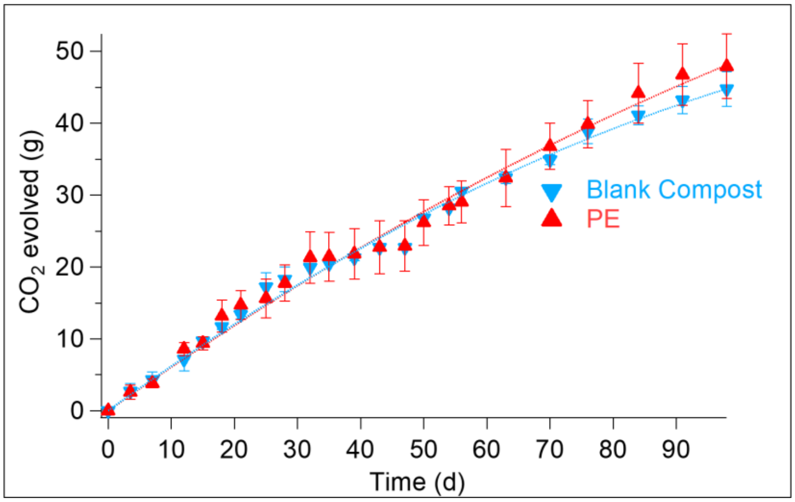

Supplementary Figure 3. Cumulative CO_2 production from the blank compost and PE buried in compost. Error bars = Standard Deviation (n=3 for PE and Cellulose, n=2 for Blank Compost).

The cellulose was mineralized at a rate of 3.87 %/d in $\Delta t_{0\text{-}18}$, reaching a 68% mineralization level after 18 days. Its mineralization rate decreased significantly to reach 1.31 and 0.19%/d in $\Delta t_{18\text{-}35}$ and $\Delta t_{35\text{-}98}$, respectively (Supplementary Figure 4). By day 98, 97% of the cellulose was mineralized. The time limit set by ASTM D 5338 is 180 days, thus even at a low mineralization rate, as observed during $\Delta t_{35\text{-}98}$, the remaining cellulose is assumed to be fully mineralized.

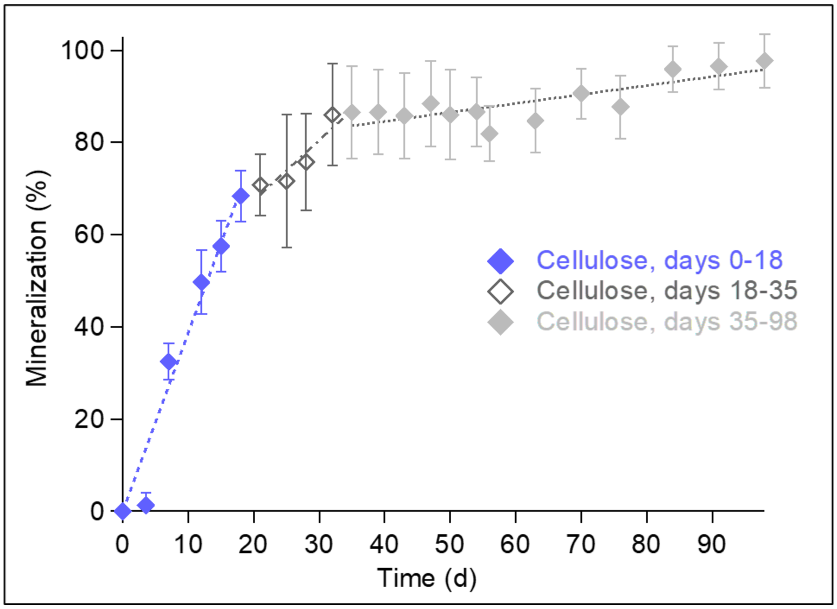

Supplementary Figure 4. The apparent rate of cellulose mineralization (Y) over 98 days of incubation was calculated for three different time periods using the following three linear regression equations:

$\Delta t_{0\text{-}18}$: $Y = 3.87 \bullet t_{0\text{-}18} + 0$ $R^2 = 0.9556$;

$\Delta t_{18\text{-}35}$: $Y = 1.31 \bullet t_{18\text{-}35} + 41.18$ $R^2 = 0.9024$;

$\Delta t_{35\text{-}98}$: $Y = 0.19 \bullet t_{35\text{-}98} + 76.84$ $R^2 = 0.6440$.

Error bars = Standard Deviation (n=3).

Mineralization

The percent of carbon by weight used to compute the mineralization levels (denominator of equation (2) of the main text) can be inferred from the molecular formula of the material, with the assumption that the tested materials are pure (absence of water or inorganic matter). In practice, the extraction process could leave water or inorganic matter (i.e. salts) as residues. Water could also be absorbed in the storage environment. Consequently, we validated such theoretical computations experimentally. The dry solids, the ash content (inorganic matter) and the percent of carbon by weight of the materials tested were measured. The ash content is the mass left after heating at 550 °C in an oxidative environment keeping such temperature until constant mass was reached. It was obtained by means of TGA, entailing a ramp of 10 °C/min until 550 °C and then an isotherm at 550 °C until the DTG was lower than 0.01 %/min, in air atmosphere. The test was run on 4 samples for SM-E (Supplementary Figure 5) and 2 samples for the other materials.

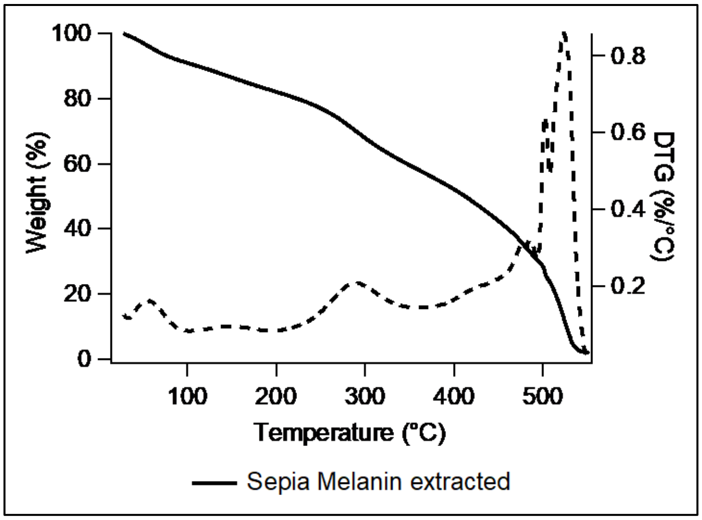

Supplementary Figure 5. Thermogravimetric analysis of Sepia Melanin extracted without any hydration treatment in air atmosphere.

Percent of C by weight was measured by Galbraith laboratories, TN, USA (carbon, hydrogen, and nitrogen determination, average of 2 tests). The amount of inorganic carbon (carbonates) for Sepia Melanin was found to be negligible (< 0.75% wt.). For Sepia Melanin, the mass of carbon present was obtained by multiplying the measured C % wt. by the organic matter, i.e., the dry solids after subtraction of ash content.

Amounts of CO_2 for complete mineralization (*CO_2 (total)* of equation (3) of main text) of the materials tested, the negative control as well as the positive control in the biodegradability test under mesophilic conditions (25 °C), test 1, and in the biodegradability test under mesophilic conditions (58 °C), test 2, are listed in Supplementary Table 3.

Considering equation (2) of the main text, the standard error of mineralization, S_e, was computed following paragraph 12.3 of ASTM D5338[14]:

$$S_e = \sqrt{\frac{s_{mat}^2}{n_1} + \frac{s_{blank}^2}{n_2}} * \frac{100}{CO_2(total)} \qquad \text{(S4)}$$

Where:

- s_{mat} is the standard deviation of the average measured mass of CO_2 evolved from the bioreactors containing the test material blended with the compost (average *CO_2 (material)* of equation (2) of the main text); n_1 is the number of such bioreactors,
- s_{blank} is the standard deviation of the average measured masses of CO_2 evolved from the bioreactors containing blank compost (average *CO_2 (blank compost)* of equation (2) of the main text)*;* n_2 is the number of bioreactors containing blank compost.
- *CO_2 (total)* is the theoretical mass of CO_2 that would evolve if all the test material were completely respired by the microorganisms (equation (3) of the main text).

Supplementary Table 3. Physicochemical properties of the materials tested.

Test Materials	Polyphenylene sulfide	Copper (II) phthalocyanine*	Polyethylene	Sepia Melanin	Cellulose
Formula	$(C_6H_4S)_n$	Cu $C_{32}H_{16}N_8$	$(CH_2)_n$	$(C_8H_7NO_2)_n$ + $(C_9H_7NO_4)_m$	$(C_6H_{10}O_5)_n$
Dry solids (wt. %)	99.64 ± 0.01	98.9 ± 0.2	100 ± 0	83.1 ± 0.3	94.8 ± 0.1
Theoretical C % in the formula (wt.)	67	67	86	N/A	44
Measured C % of dry solids (wt.)	65.66 ± 0.05	64.16 ± 0.01	N/D	66.1 ± 0.1	43.8 ± 0.1
Ash Content (wt. %)	5.2 ± 0.6	16 ± 1	0	2.1 ± 0.4	0
CO_2 if complete mineralization occurred, test 1 (g)	N/D	N/D	N/D	19	13
CO_2 if complete mineralization occurred, test 2 (g)	51	51	66	41	32

Note : The amount of Cu-Pc added was chosen taking into consideration that the dye content in the material as received was 90% by weight, with the remaining 10% being inorganic, as stated by Sigma-Aldrich. N/A, not available; N/D, not determined.

Phytotoxicity Test

A natural sandy soil (0.7% clay, 2.0% organic matter, 97.6% sand, 1.6% silt, and pH 5.5–6.0) from Valcartier (QC, Canada) was used as a non-contaminated substratum to conduct the terrestrial phytotoxicity test[15]. Prior to seeding the sandy soil, its water content was adjusted to 8.3% wt., which is 75% of its water holding capacity. Water content of compost samples, which were recovered from bioreactors at the end-point (98 d) of the biodegradability test under thermophilic conditions (58 °C), was adjusted to 40% wt. Secondly, 77 g of the wet sandy soil was mixed with approximately 23 g of compost + test material, that is a 3.3:1 weight ratio. Twenty (20) seeds of ryegrass (*Lolium perenne*) were sown in 15-cm-wide dishes (TC dish, Sarstedt #83-3903) containing 100 g of wet sandy soil with compost + test material. Such TC dishes were incubated

in sealed plastic bags to ensure maintenance of the soil moisture throughout the duration of the germination test.

The phytotoxicity test was conducted on six samples collected at the end of the composting tests and on two subsamples of wet sandy soil, refrigerator stored at 5 °C. The six compost samples are: (i) blank compost, (ii) compost + PE, (iii) compost + cellulose, (iv) compost + Sepia Melanin, (v) compost + PPS, and (vi) compost + Cu-Pc. The two soil samples are: a 100-g soil sample and a 220-g soil sample. The result of an inter-experiment control test showed that the use of 100 g or 200 g of a control soil did not influence the end results of a seedling emergence test as we report 93% seedling emergence compared with our previous works[16]. These results are statistically equivalent, therefore a 100-g soil sample can be taken as the reference for the other samples.

The phytotoxicity test was performed in a controlled (light, humidity and temperature) environmental chamber (Conviron Inc., Winnipeg, MB, Canada). Planted seeds were incubated in total darkness for the first 2 d, then exposed to a diurnal photoperiod cycle: darkness for 8 h at 20 °C and light for 16 h at 25 °C, with light intensity of 5,000 ± 500 lux. The luminosity level was measured weekly using a photometer, and the light intensity was readjusted when needed. After 19 d, seedling emergence, shoot wet mass, and shoot dry mass were measured and reported herein as test end-points. Shoots were cut just above the soil line, and fresh mass was determined immediately. Total dry mass was determined after drying the plants at 70 °C for 24 h.

Analytical Methods

Infrared (IR) spectra of the melanin samples were recorded with a resolution of 4 cm^{-1} using a Bruker Optics Vertex 70 FT-IR spectrometer equipped with DLaTGS detector. The spectra shown in Supplementary Figure 1 were recorded in transmission by dispersing the melanin powder in KBr and pressing it into 7- mm pellets using a Pike Technologies Hand Press (WI, USA). The spectrum of a pure KBr pellet was subtracted and a concave rubber band baseline correction (a single iteration) was applied. Each spectrum was normalized with respect to the highest band for the sake of comparison.

TGAs were carried out by means of a TGA 2950 thermogravimetric analyzer (TA Instruments, Inc.). The atmosphere (Ar, N_2 or air), flow of 90 cm^3/min, as well as the thermal history (ramp or isotherm), are specified for each test in the Supplementary Information.

Neutron activation analysis was performed with a SLOWPOKE nuclear reactor (Atomic Energy of Canada Limited) and a germanium semiconductor gamma-ray detector (Ortec, GEM55185). The samples were irradiated for 600 s at an average thermal neutron flux of 5×10^{11} neutrons per cm^2/s.

The analysis of the amount of C by weight was performed by Galbraith Labs, Inc., USA. A PerkinElmer 2400 Series II CHNS/O Analyzer (Governing SOP: ME-14, Analyte: Carbon, Hydrogen, Nitrogen, Range:

> 0.5 % CHN) was used. This instrument burns the sample in pure O_2 atmosphere at 920° – 980 °C under static conditions to produce combustion products of CO_2, H_2O, and NO_x. The PE-2400 automatically separates and analyzes these products in a self-integrating, steady state thermal conductivity analyzer.

The Inorganic Carbon test of Sepia Melanin extracted from cuttlefish ink was performed by Galbraith labs, Inc., USA, too. This method determines carbon dioxide, carbonic acid, bicarbonate ion, and carbonate ion. A CO_2 Coulometer Model CM 5014, UIC, Inc. was used. This method liberates CO_2 from the sample through acidification with H_2SO_4 solution and heat. A CO_2-free nitrogen gas sweeps the evolved CO_2 through a scrubber and into an absorption cell, which coulometrically titrates.

Statistical analysis

The analysis of variance (ANOVA) test was carried out to compare the means of Table 1 (Cumulative O_2 consumed and Cumulative CO_2 evolved) and of Table 2 (Plant Germination and Plant Biomass) of the main text.

The basic null hypotheses for the test are:

- H_o: mean A = mean B = …mean K
- H_1: the means are not all equal

where K = the number of independent comparison groups[17].

The ANOVA outputs (SS, df, and MS) were calculated as per equations shown in Supplementary Table 4.

The F statistic for ANOVA, F=MSB/MSE was computed and compared to the F critical value (α=0.05)[18]. If F > F critical value, the null hypothesis is rejected (the means are not equal).

Supplementary Table 4. Analysis of Variance Table

Source of Treatment	Sum of Squares (SS)	Degree of Freedom (df)	Mean Squares (MS)
Between groups	SSB = $\sum n_j(\overline{X}_j - \overline{X})^2$	k-1	MSB = $\frac{SSB}{k-1}$
Error	SSE = $\sum\sum(X - \overline{X}_j)^2$	N-k	MSE = $\frac{SSE}{N-k}$

Where :

- X is an individual observation,
- $\overline{X}_j$ is the sample mean of the jth group,
- $\overline{X}$ is the overall sample mean,
- k is the number of groups,
- N is the total number of observations.

1. <u>O_2 consumed</u>

- k=3; N=7

Supplementary Table 5. Data of O_2 consumed after 97 days (Table 1 of main text).

**O_2 consumed**	**Cellulose (Positive control) (A)**	**Sepia Melanin (Test material) (B)**	**Blank compost (Background) (C)**
1	8851	2618	2192
2	8819	2927	2197
3		2731	
**Average**	**8835**	**2759**	**2194**
**Standard Deviation**	**23**	**156**	**3**

Supplementary Table 6. Analysis of Variance (O_2 consumed).

Source of Variation	Sum of Squares (SS)	Degree of Freedom (df)	Mean Squares (MS)	F	Critical value for F
Between groups	57120889	2	28560445	2312	6.94
Error	48403	4	12101		

F > F critical value, so the null hypothesis is rejected.

2. <u>CO_2 evolved</u>

- k=6: N=16

Supplementary Table 7. Data of CO_2 evolved after 98 days (Table 1 of main text).

CO_2 evolved	Cellulose (Positive control) (A)	Sepia Melanin (B)	Polyethylene (Negative control) (C)	Poly-phenylene sulfide) (D)	Blank compost (Background) (E)	Copper (II) phthalo-cyanine (F)
1	76658	53463	52221	51913	46459	38190
2	77740	66735	48054	43512	43083	39872
3	74962		43316	45074		39507
Average	76453	60099	47863	46833	44771	39189
Standard Deviation	1400	9385	4455	4468	2387	885

Supplementary Table 8. Analysis of Variance (CO_2 evolved).

Source of Variation	Sum of Squares (SS)	Degree of Freedom (df)	Mean Squares (MS)	F	Critical value for F
Between groups	2648541360	5	529708272	29.610	3.326
Error	178892142	10	17889214		

F > F critical value, so the null hypothesis is rejected.

3. **Plant Germination**

- k= 6; N=24

Supplementary Table 9. Data of plant germination (Table 2 of main text).

Plant germination	Cellulose (Positive control) (A)	Blank compost (Background) (B)	Sepia Melanin (C)	Poly-ethylene (Negative control) (D)	Copper (II) phthalo-cyanine (E)	Poly-phenylene sulfide (F)
1	19	16	17	16	14	5
2	16	11	12	6	9	9
3	18	14	14	15	11	11
4		17	17	12		
5		19		13		
6				16		
Average	18	15	15	13	11	8
Standard Deviation	2	3	2	4	3	3

Supplementary Table 10. Analysis of Variance (plant germination).

Source of Variation	Sum of Squares (SS)	Degree of Freedom (df)	Mean Squares (MS)	F	Critical value for F
Between groups	174.4	5	34.9	3.848	2.773
Error	163.2	18	9.07		

F > F critical value, so the null hypothesis is rejected.

4. Plant Biomass

- k= 6; N=24

Supplementary Table 11. Data of plant biomass (Table 2 of main text).

Observation<br><br>Plant biomass	Cellulose<br>(Positive control)<br>(A)	Blank compost<br>(Background)<br>(B)	Sepia Melanin<br>(C)	Polyethylene<br>(Negative control)<br>(D)	Copper (II) phthalo-cyanine<br>(E)	Poly-phenylene sulfide<br>(F)
1	136	77	105	97	74	6
2	93	50	45	20	12	17
3	110	97	74	47	64	18
4		73	82	40		
5		124		36		
6				46		
Average	113	84	77	48	50	14
Standard Deviation	21	28	24	26	33	6

Supplementary Table 12. Analysis of Variance (plant biomass).

Source of Variation	Sum of Squares (SS)	Degree of Freedom (df)	Mean Squares (MS)	F	Critical value for F
Between groups	19655.2	5	3931.0	6.1	2.773
Error	11578.8	18	643.1		

F > F critical value, so the null hypothesis is rejected.

Supplementary References

1. Magarelli, M., Passamonti, P. & Renieri, C. Purification , characterization and analysis of sepia melanin from commercial sepia ink (Sepia Officinalis) Purificación , caracterización y análisis de la melanina de sepia a partir de la tinta de sepia (Sepia Officinalis) Resumen. *Rev. CES Med. Vet. y Zootec.* **5**, 18–28 (2010).
2. Araujo, M., Xavier, J. R., Nunes, C. D., Vaz, P. D. & Humanes, M. Marine sponge melanin: A new source of an old biopolymer. *Struct. Chem.* **23**, 115–122 (2012).
3. Centeno, S. A. & Shamir, J. Surface enhanced Raman scattering (SERS) and FTIR characterization of the sepia melanin pigment used in works of art. *J. Mol. Struct.* **873**, 149–159 (2008).
4. Hong, L. & Simon, J. D. Current understanding of the binding sites, capacity, affinity, and biological significance of metals in melanin. *J. Phys. Chem. B* **111**, 7938–7947 (2007).
5. Albano, L. G. *et al.* Novel insights on the physicochemical properties of eumelanins and their DMSO derivatives. *Polym. Int.* **26**, 19007–19013 (2016).
6. Young, J. C., Garner, W. & Clark, J. W. An Improved Apparatus for Biochemical Oxygen Demand. *Anal. Chem.* **20**, 1964 (1964).
7. Young, J. C. & Cowan, R. M. *Respirometry for Environmental Science and Engineering*. (SJ Enterprises, 2004).
8. Miltner, A., Richnow, H.-H., Kopinke, F.-D. & Kästner, M. Incorporation of carbon originating from CO 2 into different compounds of soil microbial biomass and soil organic matter†. *Isotopes Environ. Health Stud.* **41**, 135–140 (2005).
9. Miltner, A. *et al.* Non-phototrophic CO 2 fixation by soil microorganisms. *Plant Soil* **269**, 193–203 (2005).
10. Yuan, H., Ge, T., Chen, C., O'Donnell, A. G. & Wu, J. Significant Role for Microbial Autotrophy in the Sequestration of Soil Carbon. *Appl. Environ. Microbiol.* **78**, 2328–2336 (2012).
11. Rho, D. & André, G. Growth and stoichiometry of a Catharanthus roseus cell suspension culture grown under nitrogen-limiting conditions. *Biotechnol. Bioeng.* **38**, 579–587 (1991).
12. Magalhães, A. M. T., Shea, P. J., Jawson, M. D., Wicklund, E. A. & Nelson, D. W. Practical Simulation of Composting in the Laboratory. *Waste Manag. Res.* **11**, 143–154 (1993).
13. ASTM D6400-12, Standard Specification for Labeling of Plastics Designed to be Aerobically Composted in Municipal or Industrial Facilities. *ASTM International* **1**, 1–3 (2012).
14. ASTM D5338- 2015, Standard Test Method for Determining Aerobic Biodegradation of Plastic Materials Under Controlled Composting Conditions. (2015). doi:10.1520/D5338-15.2
15. Dodard, S. G. *et al.* Ecotoxicological assessment of a high energetic and insensitive munitions compound: 2,4-Dinitroanisole (DNAN). *J. Hazard. Mater.* **262**, 143–150 (2013).
16. Rocheleau, S. *et al.* Phytotoxicity and uptake of nitroglycerin in a natural sandy loam soil. *Sci. Total Environ.* **409**, 5284–5291 (2011).
17. Miller, R. G. J. *Beyond ANOVA: Basics of Applied Statistics*. (CRC Press, 1997).
18. Ahrens, H. Pearson, E. S., Ho. O. Hartley: Biometrika Tables for Statisticians, Vol. 2. Cambridge University Press, London 1972. XVIII, 385 S., £ 5.00. *Biom. Z.* **16**, 291–291 (1974).
